# DNA methyltransferase 3B plays a protective role against hepatocarcinogenesis caused by chronic inflammation via maintaining mitochondrial homeostasis

**DOI:** 10.1038/s41598-020-78151-2

**Published:** 2020-12-04

**Authors:** Eriko Iguchi, Atsushi Takai, Haruhiko Takeda, Ken Kumagai, Soichi Arasawa, Yuji Eso, Takahiro Shimizu, Yoshihide Ueda, Hiroyuki Marusawa, Hiroshi Seno

**Affiliations:** 1 Department of Gastroenterology and Hepatology, Graduate School of Medicine, Kyoto University, 54 Kawahara-cho, Shogoin, Sakyo-ku, Kyoto, 606-8507 Japan; 2 Department of Gastroenterology and Hepatology, Graduate School of Medicine, Kobe University, Hyogo, Japan; 3 Department of Gastroenterology and Hepatology, Osaka Red Cross Hospital, Osaka, Japan

**Keywords:** Hepatocellular carcinoma, DNA methylation, Mitochondria, Hepatitis

## Abstract

Most hepatocellular carcinomas (HCCs) develop on the basis of chronic hepatitis, but the mechanism of epigenetic regulation in inflammatory hepatocarcinogenesis has yet to be elucidated. Among de novo DNA methyltransferases (DNMTs), DNMT3B has lately been reported to act specifically on actively transcribed genes, suggesting the possibility that it plays a role in the pathogenesis of cancer. We confirmed that DNMT3B isoforms lacking its catalytic domain were highly expressed in HCCs compared with non-tumorous liver tissue. To elucidate the role of DNMT3B in hepatocarcinogenesis, we generated a genetically engineered mouse model with hepatocyte-specific *Dnmt3b* deletion. The liver of the *Dnmt3b*-deficient mice exhibited an exacerbation of thioacetamide-induced hepatitis, progression of liver fibrosis and a higher incidence of HCC compared with the liver of the control mice. Whole-genome bisulfite sequencing verified a lower CG methylation level in the *Dnmt3b*-deficient liver, demonstrating differentially methylated regions throughout the genome. Transcriptome analysis revealed decreased expression of genes related to oxidative phosphorylation in the *Dnmt3b*-deficient liver. Moreover, primary hepatocytes isolated from the *Dnmt3b*-deficient mice showed reduced mitochondrial respiratory capacity, leading to the enhancement of oxidative stress in the liver tissue. Our findings suggest the protective role of DNMT3B against chronic inflammation and HCC development via maintaining mitochondrial homeostasis.

## Introduction

Hepatocellular carcinoma (HCC) is one of the most common human malignancies in the world^1^. Most HCCs develop on the basis of chronic hepatitis and cirrhosis, mainly caused by hepatitis virus infection and steatohepatitis. Similar to other cancers, HCCs exhibit abnormal DNA methylation^2–4^. Aberrant hypermethylation in CpG islands has been detected not only in advanced HCCs but also in early HCCs and precancerous nodules, such as dysplastic nodules^5–7^. Additionally, the surrounding non-cancerous liver tissue exhibits an abnormal DNA methylation profile^6^; the alteration of the DNA methylation pattern varies with the etiology and stage of the liver disease^8,9^. Particularly, hypermethylation in the promoter regions of several tumor suppressor genes results in HCC development^10,11^. Thus, dysregulation of DNA methylation, especially at the promoter CpG region, is strongly associated with the multistep hepatocarcinogenesis process. In contrast, hypomethylation at gene bodies is generally observed in various cancers. The significance of methylation alterations at the gene body, however, remains to be understood.

DNA methylation is mediated by DNA methyltransferases (DNMTs). Three major DNMTs in mammals are DNMT1, DNMT3A, and DNMT3B. While DNMT1 plays an important role in the maintenance of methylation after DNA replication, DNMT3A and DNMT3B contribute to de novo DNA methylation^12,13^. Both DNMT3A and DNMT3B are expressed during embryo development, they establish DNA methylation patterns, and their expression levels decrease with biological development into adulthood. DNA methylation patterns are mainly preserved by DNMT1 in the adult tissue, while DNMT3A and DNMT3B are involved in methylation maintenance along with DNMT1^14^. Dysregulation of de novo DNMTs has been observed in several types of tumors. For example, loss-of-function mutations in DNMT3A have been observed in hematologic malignancies, such as acute myeloid leukemia, myelodysplastic syndrome, and chronic myelomonocytic leukemia, and they are associated with treatment resistance^15^. On the other hand, overexpression of DNMTs has also been observed in various cancers. DNMT3B is highly expressed in breast, colon, and prostate cancers^16–18^. Both DNMT3A and DNMT3B have been reported to be highly expressed in liver cancer than in non-cancerous tissue^19^. Interestingly, Saito et al. reported that a splice variant of DNMT3B, DNMT3B4, which lacks conserved methyltransferase motifs, was highly expressed in HCCs and showed that overexpression of DNMT3B4 resulted in DNA hypomethylation in 293 cells^20^. However, it still remains to be elucidated how the dysregulated DNMT3B contributes to HCC development in patients with chronic hepatitis.

DNMT3A and DNMT3B have a common catalytic domain in the carboxyl-terminus and a PWWP (Pro-Trp-Trp-Pro) motif in the amino terminus. The PWWP domain in DNMTs is important for localizing these enzymes at the heterochromatin region. While both DNMT3A and DNMT3B preferentially target CpG-rich sequences, DNMT3A exhibits a higher DNA methylation activity in naked DNA compared with DNMT3B; however, only DNMT3B can methylate DNA in the nucleosome core region^21^. DNMT3B selectively binds to the bodies of transcribed genes and contributes to their preferential methylation^22^, and DNMT3B-dependent intragenic DNA methylation plays an important role in protecting the gene body from spurious initiation of transcription^23^. In the current study, we focused on the role of DNMT3B in regulating the DNA methylation pattern on the development of chronic hepatitis and HCC. *Dnmt3b* deletion in the whole body produces embryonic lethality in mice^24^; therefore, the functional role of *Dnmt3b *in vivo has not been well elucidated. We have generated a novel mouse model in which the *Dnmt3b* gene is specifically deleted in hepatocytes and have revealed that DNMT3B plays an important role in protecting the liver tissue from the exacerbation of hepatitis and hepatocarcinogenesis.

## Results

### *DNMT3B* lacking its catalytic domain is overexpressed in hepatocellular carcinoma tissues

**Figure 1:**
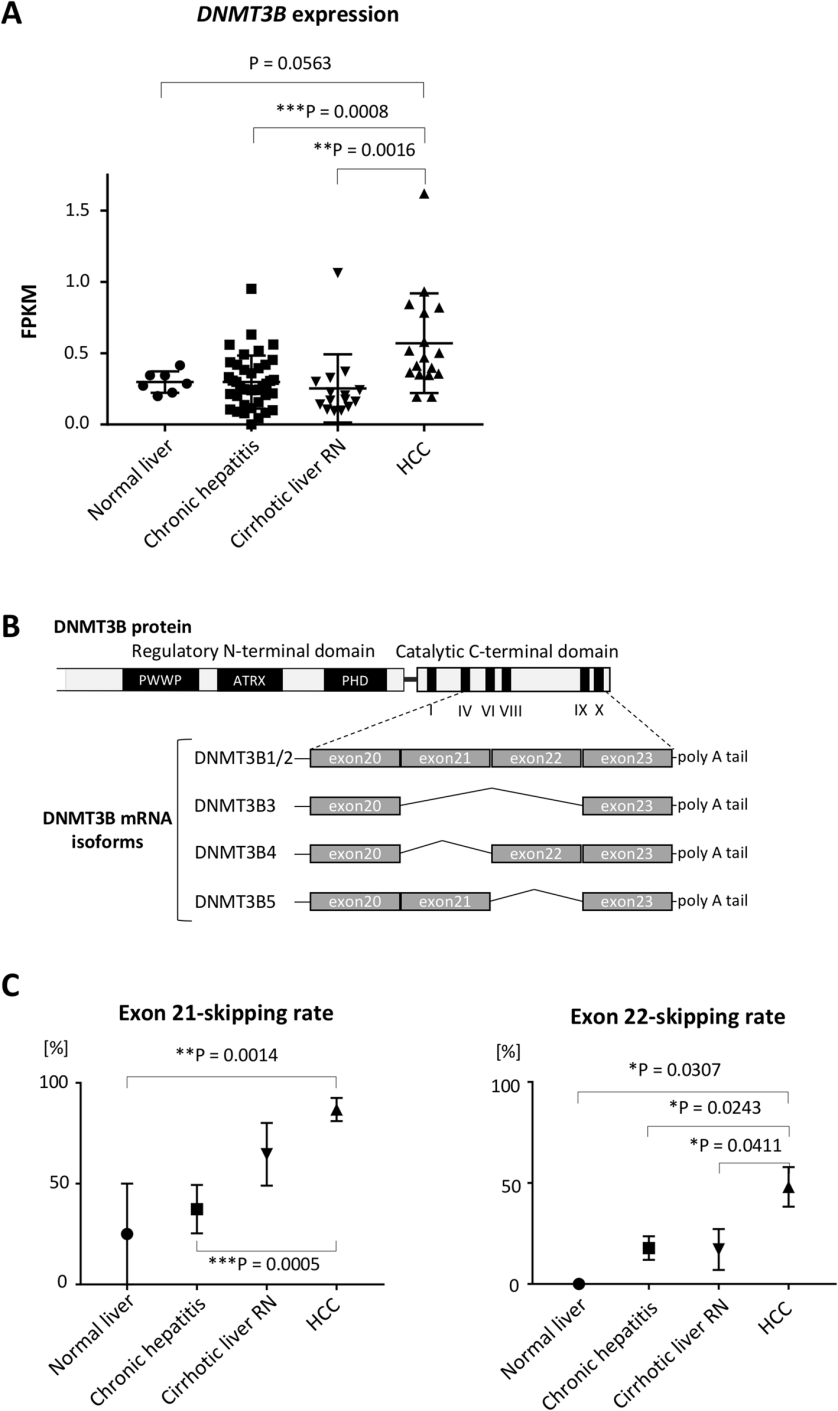
*DNMT3B* lacking catalytic domain is highly expressed in regenerative nodules (RN) and hepatocellular carcinoma (HCC) tissues. (**A**) *DNMT3B* gene expression level in surgical specimens, represented as fragments per kilobase million (FPKM), based on RNAseq data. N = 7 for normal liver tissue samples, 40 for chronic hepatitis tissue samples, 15 for RN samples and 17 for HCC tissue samples. P values were determined by Tukey’s multiple comparison test. Data are presented as the mean ± s.d. (**B**) Scheme of DNMT3B protein structure and representative DNMT3B mRNA isoforms. (**C**) Estimation from RNAseq data of the skipping rate of exon 21 or 22 in *DNMT3B* mRNA expressed in liver tissues. N = 7 for normal liver tissue samples, 15 for chronic hepatitis tissue samples, 12 for RN samples and 17 for HCC tissue samples. P values were determined by Tukey’s multiple comparison test. Data are presented as the mean ± s.e.m. Images in (**A**) and (**C**) were made on GraphPad Prism ver 7.00.

First, we examined the expression level of *DNMT3B* in normal liver tissues, chronic hepatitis tissues, cirrhotic liver regenerative nodules (RNs) and HCC tissues. We used RNA sequencing (RNAseq) data from 17 HCC tissue samples, 15 RN samples and 40 hepatitis-C-associated chronic hepatitis tissue samples, along with 7 normal liver tissues from donors for living-donor liver transplantation as a control. Consistent with the previous reports^19,20^, HCC tissues showed a significantly higher level of *DNMT3B* expression compared with other non-cancerous tissues (Fig. 1A). Further analysis of these RNAseq data showed that a large proportion of the expressed DNMT3B in HCC tissues did not have exons 21 and/or 22, which encode a C-terminal catalytic domain (Fig. 1B,C)^12,25,26^. Notably, exon 21 tended to be skipped not only in HCC tissues but also in cirrhotic liver RNs (Fig. 1C, left panel). These data suggest that the dysregulation of *DNMT3B* gradually occurs during the progression of liver cirrhosis and is associated with HCC development.

### Hepatocyte-specific *Dnmt3b*-deficient mice exhibit normal development

**Figure 2:**
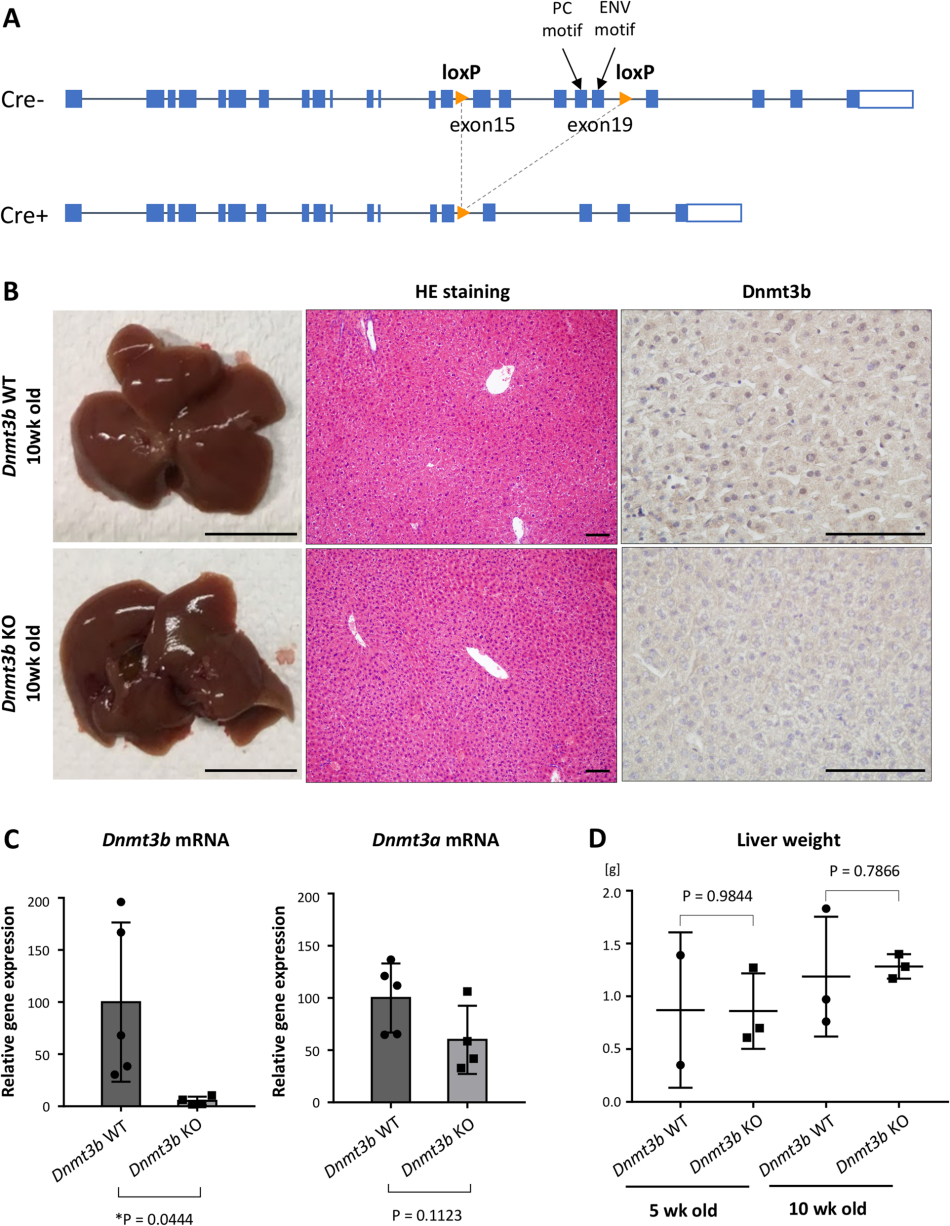
Hepatocyte-specific *Dnmt3b*-deficient (KO) mice show normal liver growth. (**A**) Location of loxP sequences that are designed to generate *Dnmt3b*-deficient mice, shown schematically, on *Dnmt3b* gene regions based on the NCBI gene database. (**B**) (Left panels) Representative macroscopic appearance of the liver collected from a *Dnmt3b*-WT mouse and KO mouse at 10 weeks of age. Scale bar, 1 cm. Representative hematoxylin and eosin (HE) staining (Middle panels, 100 × magnification) and *Dnmt3b* immunohistochemical staining (Right panels, 400 × magnification) of liver sections from a *Dnmt3b*-WT mouse and KO mouse at 10 weeks of age. Scale bar, 100 µm. (**C**) mRNA expression level of *Dnmt3b* and *Dnmt3a* in liver tissues from *Dnmt3b*-WT and KO mice at 6 weeks of age. N = 5 for WT mice, and N = 4 for KO mice. (**D**) Liver weight of *Dnmt3b*-WT mice and KO mice at 5 or 10 weeks of age. At 5 weeks of age, N = 2 for WT mice, and N = 3 for KO mice. At 10 weeks of age, N = 3 for both genotypes. P values were determined using the two-tailed *t* test in both (**B**) and (**C**). Data are presented as the mean ± s.d. Images in (**C**) and (**D**) were made on GraphPad Prism ver 7.00.

To clarify the function of *Dnmt3b* in the liver in vivo, we generated hepatocyte-specific *Dnmt3b*-deficient mice using the Cre-loxP system (*Alb-Cre*; *Dnmt3bfl/fl*). Among 23 exons of the *Dnmt3b* gene, exons 15–19 were floxed and designed to be deleted by Cre recombinase so that the catalytic activity could be lost by deleting the highly conserved PC motif and ENV motif, which are located on exons 18 and 19, respectively (Fig. 2A)^12^.

Hepatocyte-specific *Dnmt3b*-deficient mice showed normal systemic development, and their growth was almost equivalent to that of control mice until 60 weeks of age (Supplementary Fig. S1A, left panel). Knockout of *Dnmt3b* was confirmed both at the protein level (Fig. 2B, right panels) and at the mRNA level (Fig. 2C, left) by immunohistochemistry and quantitative real-time polymerase chain reaction (RT-qPCR), respectively, while *Dnmt3a* was not downregulated significantly under the *Dnmt3b*-deficient condition (Fig. 2C, right). The macroscopic appearance and weight of the liver in the *Dnmt3b*-deficient mice was comparable with that in the control mice (Fig. 2B, left panels; Fig. 2D; Supplementary Fig. S1A, right panel). In contrast to the previous report that showed the total deletion of *Dnmt3b* in the whole body resulted in embryonic lethality with liver hypotrophy^24^, our histological examination revealed that there was no difference in the liver tissue between the control mice and *Dnmt3b*-deficient mice both at 10 weeks (Fig. 2B, middle panels) and 60 weeks of age (Supplementary Fig. S1B).

### TAA-induced hepatitis is exacerbated in *Dnmt3b*-deficent mice and lead to the accelerated liver fibrosis and development of HCC

**Figure 3:**
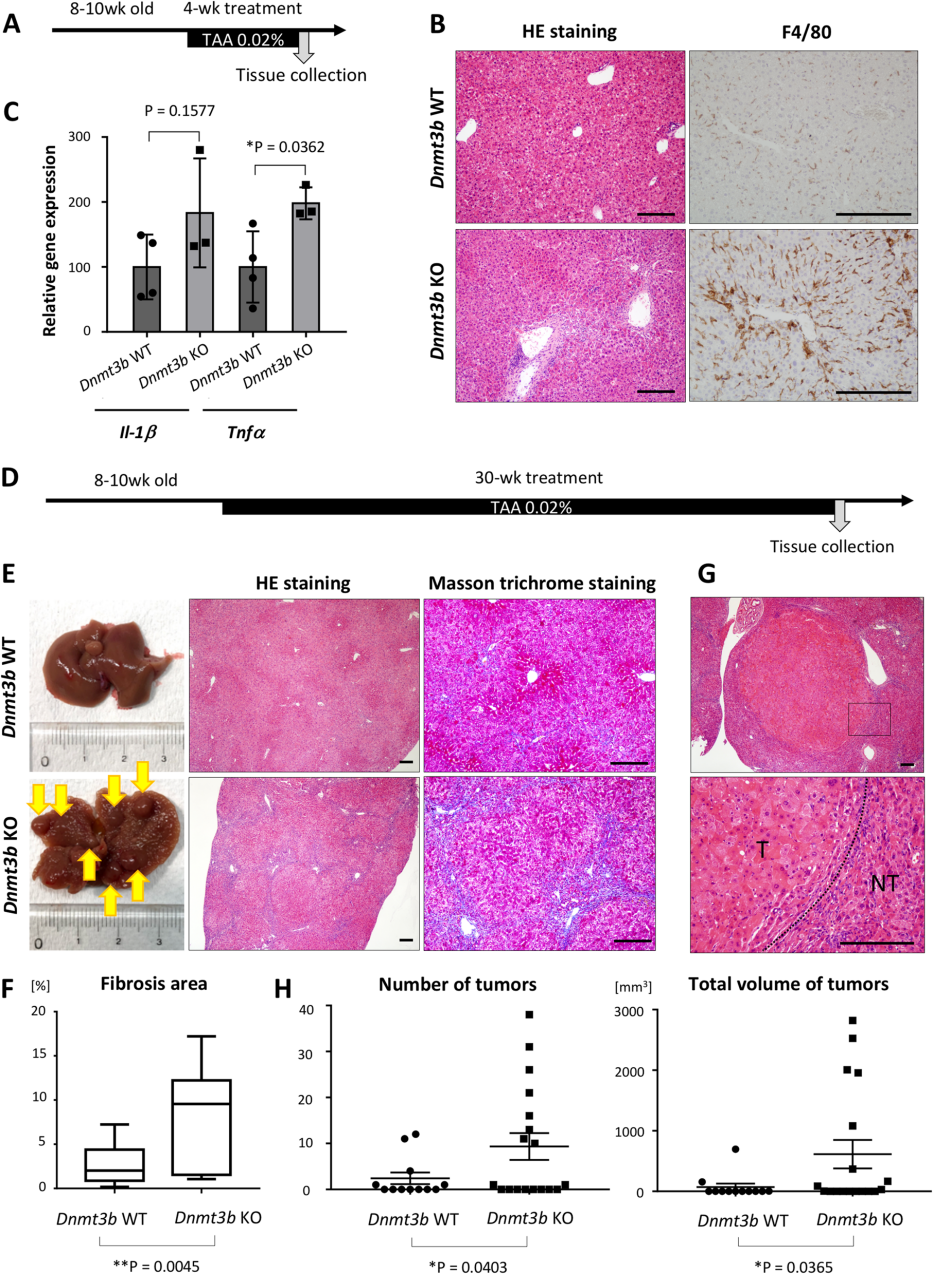
Thioacetamide (TAA)-induced hepatitis was enhanced in *Dnmt3b*-deficient (KO) mice and led to the accelerated liver fibrosis and development of liver tumors. (**A**) Protocol for TAA treatment in drinking water. (**B**) Representative HE staining (Left panels) and F4/80 immunohistochemical staining (Right panels) of liver sections from a *Dnmt3b*-WT mouse and KO mouse after 4 weeks of 0.02% TAA treatment. Scale bar, 200 µm. (**C**) mRNA expression of *Il-1β* and *Tnfα* in liver tissues from *Dnmt3b*-WT mice and KO mice after 4 weeks of 0.02% TAA treatment. N = 4 for WT, and N = 3 for KO. P values were determined using the two-tailed *t* test. Data are presented as the mean ± s.d. (**D**) Protocol for TAA treatment in drinking water. (**E**) (Left panels) Representative macroscopic appearance of liver collected from a *Dnmt3b*-WT mouse and a KO mouse after 30 weeks of 0.02% TAA treatment. Representative HE staining (Middle panels) and Masson trichrome staining (Right panels) of liver sections from a *Dnmt3b*-WT mouse and KO mouse after 30 weeks of 0.02% TAA treatment. Scale bar, 200 µm. (**F**) The percentage of fibrosis area quantified based on Masson trichrome staining in *Dnmt3b*-WT mice and KO mice after 30 weeks of 0.02% TAA treatment. N = 12 for WT, and N = 18 for KO. The P value was determined using the two-tailed *t* test. Whiskers show the minimum and the maximum. (**G**) Representative HE-stained image of liver tumors that developed in *Dnmt3b*-KO mice. The rectangular area marked in the top panel is magnified in the bottom panel. Tumor tissue was clearly separated by a capsule from non-tumor tissue (dotted line). T, tumor tissue; NT, non-tumor tissue. Scale bar, 200 µm. (H) The number (left) and the total volume (right) of tumors detected in the liver from *Dnmt3b*-WT mice and KO mice after 30 weeks of 0.02% TAA treatment. The liver was sliced into 2 mm thick sections and examined. Tumor volume was calculated by using the formula: Tumor volume = Length × Width^2^/2. N = 12 for WT, and N = 18 for KO. P values were determined using two-tailed *t* test with Welch’s correction. Data are presented as the mean ± s.e.m. Images in (**C**), (**F**) and (**H**) were made on GraphPad Prism ver 7.00.

To explore the role of *Dnmt3b* in the liver tissue under chronic inflammation, we induced hepatitis in *Dnmt3b*-deficient mice by adding 0.02% thioacetamide (TAA) in drinking water^27,28^ for four weeks and collected the liver tissues for further examination (Fig. 3A). Remarkably, the *Dnmt3b*-deficient mouse liver showed severe hepatitis especially around the Glisson sheath as compared to the liver of *Dnmt3b*-wild type (WT) mice (Fig. 3B, left panels), with massive infiltration of macrophages (Fig. 3B, right panels) as well as the increased recruitment of neutrophils and lymphocytes (Supplementary Fig. S2A). The number of Ki67-positive cells was increased (Supplementary Fig. S2B), and the expression levels of *Il1β* and *Tnfα* were elevated in the *Dnmt3b*-deficient mouse liver (Fig. 3C). These findings indicate that TAA-induced hepatitis is exacerbated in *Dnmt3b*-deficient mice.

To determine the role of *Dnmt3b* in long-term chronic hepatitis, we extended the duration of TAA treatment (0.02% in drinking water) up to 30 weeks (Fig. 3D). The *Dnmt3b*-WT mice (N = 12) and deficient mice (N = 18) showed similar body, liver, and spleen weights (Supplementary Fig. S3A). Pathological examination, however, revealed that liver fibrosis was more evident in the *Dnmt3b*-deficient mice (Fig. 3E, middle panels). Masson trichrome staining clearly shows a significant increase in bridging fibrosis in the *Dnmt3b*-deficient liver, indicating the progression of liver cirrhosis (Fig. 3E, right panels, Fig. 3F). Moreover, the *Dnmt3b*-deficient mice developed multiple liver tumors more frequently than *Dnmt3b*-WT mice (Fig. 3E, left panels). Pathologically, most of these tumors were typical well-differentiated HCCs, exhibiting capsule formation (Fig. 3G). Consistent with human HCC, some liver tumors expressed α-fetoprotein, a representative tumor marker for HCC (Supplementary Fig. S3B). The number and total volume of tumors were significantly higher in the *Dnmt3b*-deficient mice than in the WT mice (Fig. 3H). These data suggest that TAA-induced chronic hepatitis was enhanced by *Dnmt3b* loss, leading to accelerated fibrosis and carcinogenesis, which mimics the course of hepatocarcinogenesis in humans. *Dnmt3b* could exert a protective effect against inflammation-associated hepatocarcinogenesis.

### *Dnmt3b*-deficient liver has lower methylation levels in the whole genome with differentially methylated regions mainly distributed to the gene body

**Figure 4:**
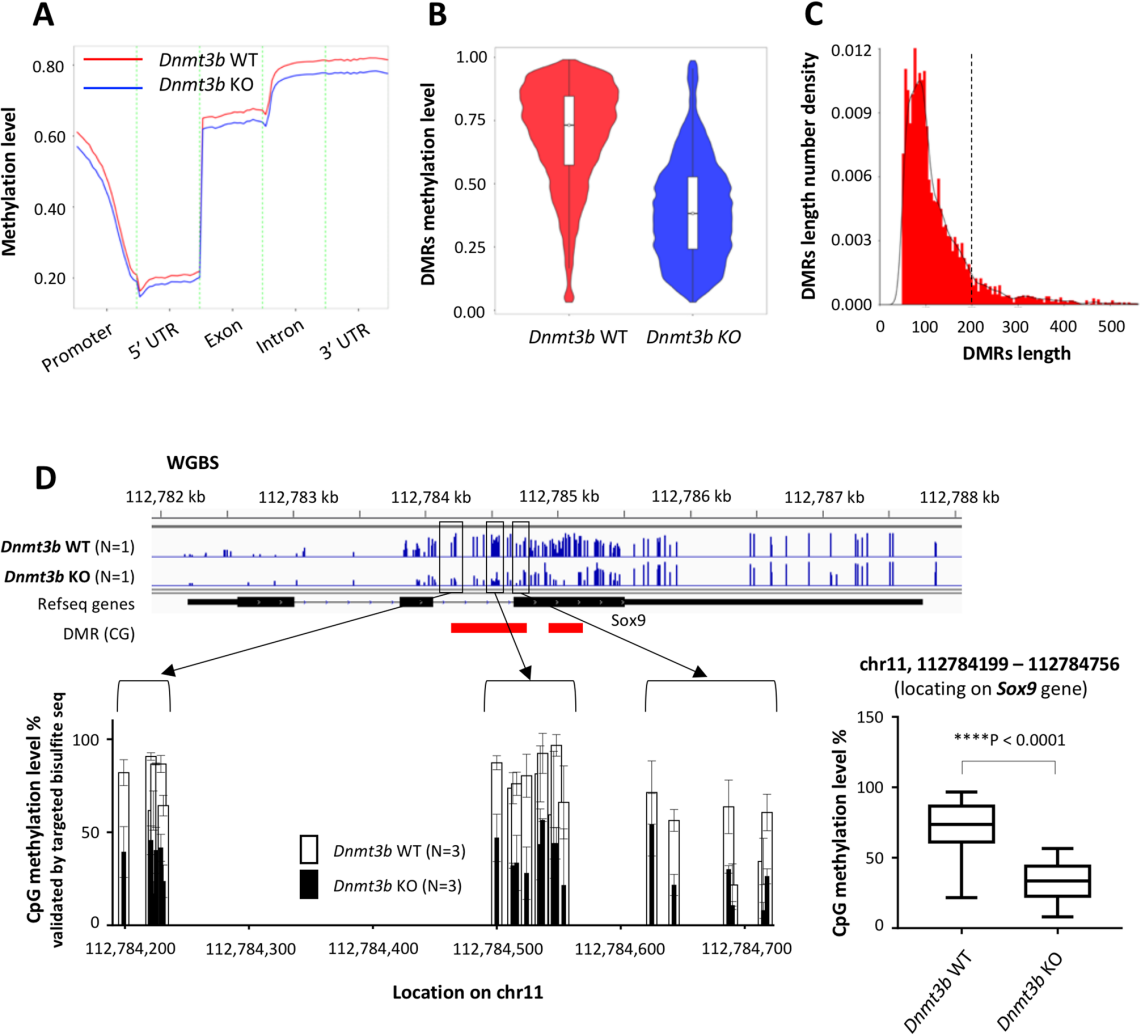
Hypomethylated CpG sites were frequently detected in the whole genome of the *Dnmt3b*-deficient (KO) liver. (**A**) Distribution of methylation levels in the CG context on gene functional elements. Each region of each gene was equally divided into 50 bins; subsequently, the C-site levels of the corresponding bins of all regions were averaged. Promoter was defined as a region of 2 kb length located upstream of the TSS site. (**B**) The violin plot indicates the distribution of methylation levels of differentially-methylated regions (DMRs) in the CG context. (**C**) Distribution of the DMR length. The dotted line indicates a length of 200 bp, DMRs longer than those, which we determined as the target of analysis. (**D**) (top) CpG methylation level in *Sox9* gene obtained from whole-genome bisulfite sequencing (WGBS). DMRs are indicated by red rectangles. (bottom, left) CpG methylation level at each CpG site in one of the *Sox9* DMRs calculated by targeted bisulfite sequencing, conducted on the loci where primers could be designed, shown by black rectangles. Data are presented as the mean ± s.d. N = 3 for both genotypes. (Bottom, right) The overall methylation level in this DMR. Whiskers show the minimum and the maximum. The P value was determined using the two-tailed paired *t* test. Images in (**D**) were made on GraphPad Prism ver 7.00.

Next, we performed whole-genome bisulfite sequencing (WGBS) to reveal the comprehensive methylome change induced by depletion of the *Dnmt3b* gene. DNA samples were extracted from non-cancerous liver tissues of 10-week-old *Dnmt3b*-WT mice and deficient mice. The methylated-CG (mCG) percentage in the whole genome was 76.94% and 66.35% in the *Dnmt3b*-WT and *Dnmt3b*-deficient mice, respectively, indicating that the overall level of DNA cytosine methylation was lowered in the *Dnmt3b*-deficient liver (Fig. 4A and Supplementary Fig. S4A). The difference in methylation level appears to be distributed almost equally in all the functional gene elements: promoters, 5′UTR, exons, introns, and 3′UTR (Fig. 4A).

Focusing on methylation in the CG context, 4192 differentially methylated regions (DMRs) were identified throughout the genome (Supplementary Fig. S4B). The methylation level in these CG-context DMRs was evidently decreased in the *Dnmt3b*-deficient samples (Fig. 4B). Among 4192 DMRs with lengths ranging from 51 to 1695 bps, we extracted 389 DMRs in the CG-context spanning longer than 200 bps (Fig. 4C, Supplementary Table S1) based on the definition of the CpG island length^29^. As much as 57.6% of CpGs included in the 389 DMRs were located on the gene body, i.e., in the exons or introns (Supplementary Fig. S4C). We focused on 363 DMRs on 424 genes, which exhibited a lower mCG level in the *Dnmt3b*-deficient mice, out of the 389 DMRs for the further analysis. Functional annotation analysis using DAVID Bioinformatics Resources 6.8 (Supplementary Table S2) revealed that an annotation cluster related to transcription regulation was highly enriched (group enrichment score = 1.865) (Supplementary Table S3).

In order to validate the result of WGBS performed using one individual of *Dnmt3b*-WT and deficient mouse, we conducted targeted bisulfite sequencing on several DMRs locating on genes included in the cluster of transcription regulation, such as *Sox9*, *Sall1* and *Mdk*, using DNA samples from three mice of each genotype (Fig. 4D and Supplementary Fig. S5A,B). Consistent with the WGBS results, the methylation level at each CpG site was considerably decreased, resulting in a significant decrease in the total methylation level in each DMR, e.g., from 71.10 to 33.73% in one DMR located on *Sox9* gene (Fig. 4D).

To learn the correlation between the methylation alteration and the expression change, we exploited RNAseq data of RNA samples from non-cancerous liver tissue of mice free from TAA treatment (N = 4 in each genotype). Intriguingly, the total length of DMR within a gene body had a weak positive correlation with the expression fold change of the gene where the DMR was located (Supplementary Fig. S6). These results suggest the possibility that the gene expression change depends on the length of DMR in the gene body.

### Oxidative phosphorylation is the top gene set downregulated in *Dnmt3b-*deficient liver

**Figure 5:**
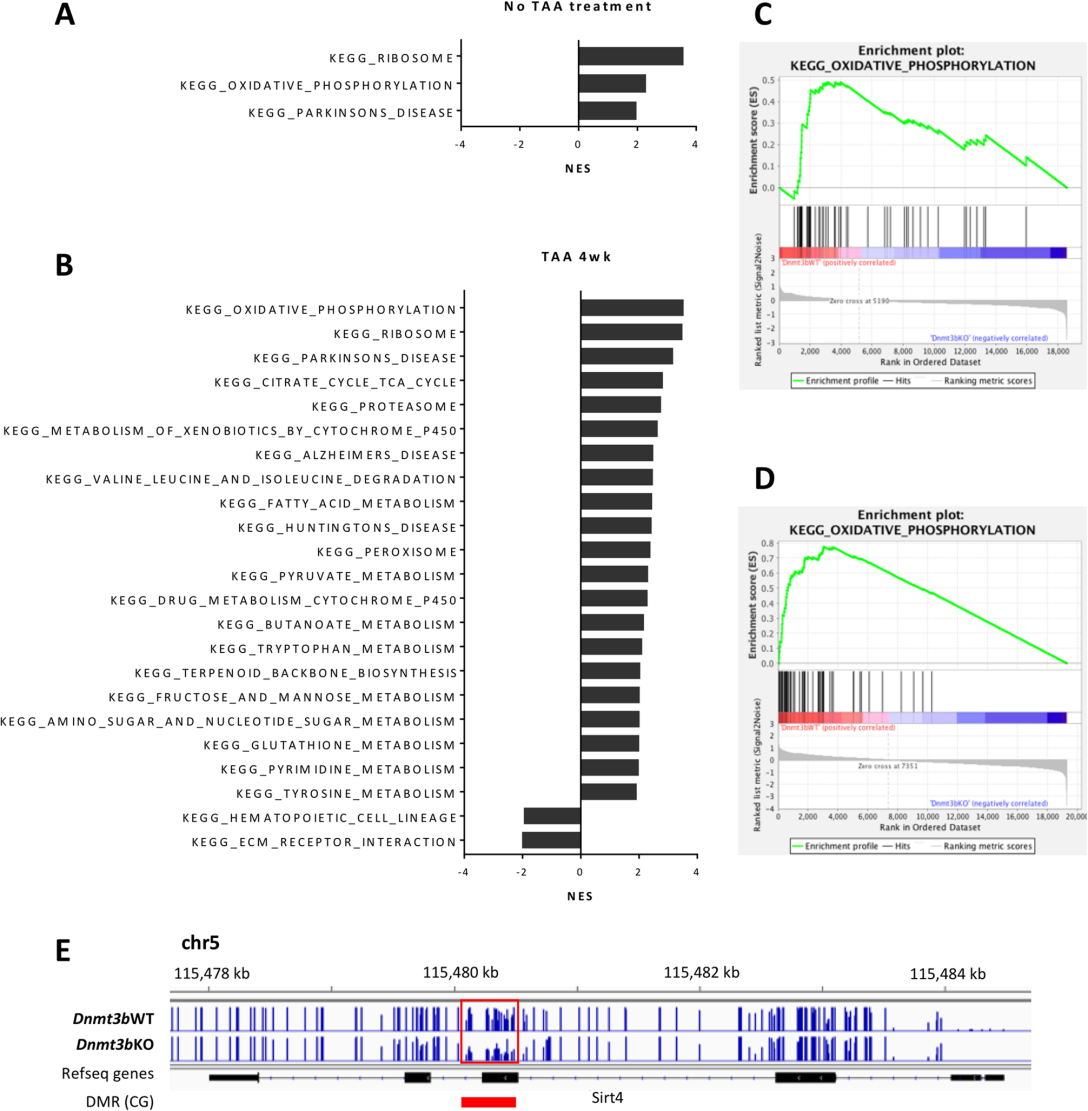
Genes related to oxidative phosphorylation are downregulated in *Dnmt3b*-deficient (KO) liver. (**A**) Normalized enrichment score (NES) of significantly enriched KEGG gene sets through gene set enrichment analysis (GSEA) of four *Dnmt3b*-WT mice and four KO mice with no thioacetamide (TAA) treatment. Enrichment plot for the gene set KEGG_OXIDATIVE_PHOSPHORYLATION is shown in (**C**). (**B**) NES of significantly enriched KEGG gene sets through GSEA of four *Dnmt3b*-WT mice and four KO mice treated with TAA for 4 weeks. Enrichment plot for the gene set KEGG_OXIDATIVE_PHOSPHORYLATION is shown in (**D**). FWER P-value cutoff = 0.05 and FDR Q-value cutoff = 0.01 in both (**A**) and (**B**). (**E**) IGV screenshot of the *Sirt4* gene demonstrating CpG methylation level obtained from WGBS and a differentially methylated regions (DMR) on the gene body, indicated by a red rectangle.

To elucidate molecular pathways involved in hepatitis and hepatocarcinogenesis that are enhanced by *Dnmt3b* depletion, we performed gene set enrichment analysis (GSEA) using RNAseq data. GSEA revealed that two gene sets, oxidative phosphorylation and ribosomal biogenesis, were significantly downregulated in the *Dnmt3b*-deficient liver without TAA treatment (Fig. 5A,C, Supplementary Table S4). In contrast, TAA-induced hepatitis considerably changed the gene expression profile in the *Dnmt3b*-deficient liver by inducing significant enrichment in 21 gene sets (Fig. 5B,D, Supplementary Table S5). Interestingly, the two gene sets enriched without TAA treatment ranked on top of the list under TAA treatment as well (Fig. 5A,B, Supplementary Tables S4 and S5). These data suggest that the expression change in the gene sets of oxidative phosphorylation and ribosomal biogenesis were related to *Dnmt3b* loss.

Focusing on the oxidative phosphorylation pathway, various genes involved in the constitution of mitochondrial complexes, including NADH: ubiquinone oxidoreductase subunit (*Nduf*) genes, were found to be downregulated in the *Dnmt3b*-deficient mouse liver (Supplementary Fig. S7A). Interestingly, we observed a DMR in the gene body of *Sirt4* with decreased methylation levels in the *Dnmt3b*-deficient liver (Fig. 5E), which was validated by targeted bisulfite sequencing using samples from three mice of each genotype (Supplementary Fig. S7B), accompanied by the upregulation of gene expression (Supplementary Fig. S7C). *Sirt4* is known to play an important role in regulating mitochondrial metabolism^30^. These data suggest that mitochondrial dysfunction caused by the dysregulation of DNA methylation could enhance hepatitis in TAA-treated *Dnmt3b*-deficient mice.

### Oxidative stress is enhanced in *Dnmt3b*-deficient liver tissue due to the reduction of mitochondrial respiratory capacity

**Figure 6:**
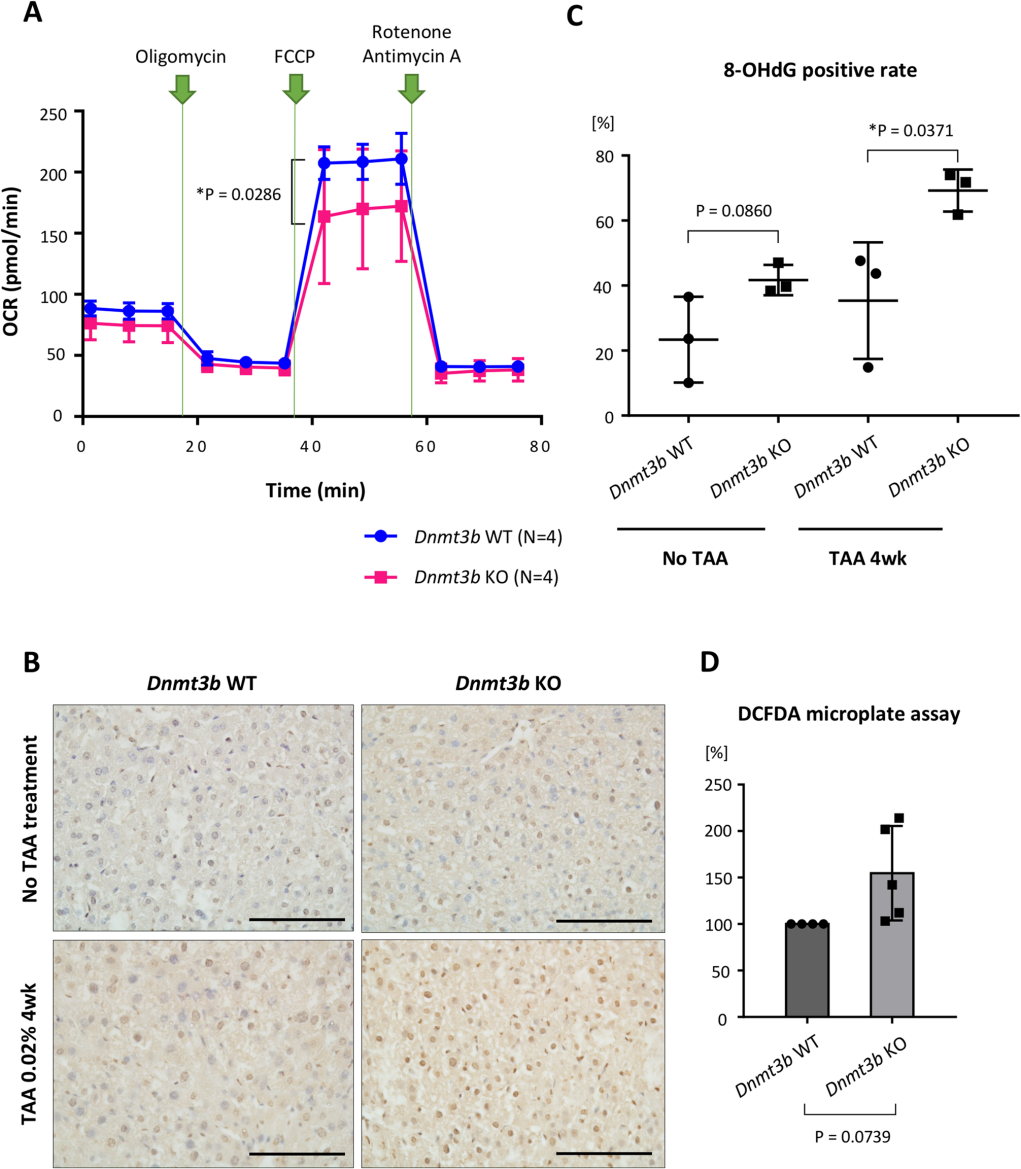
*Dnmt3b* depletion diminishes mitochondrial respiratory capacity and results in ROS accumulation in hepatocytes. (**A**) The oxygen consumption rate (OCR), as measured using the Seahorse XF Cell Mito Stress Test, is presented. The difference between maximal respiration and basal respiration is the spare respiratory capacity. The P value was determined using two-way ANOVA and Sidak’s multiple comparisons test. Data are presented as the mean ± s.d. (**B**) Representative images of 8-hydroxy-2′-deoxyguanosine (8-OHdG) immunohistochemical staining of liver sections from *Dnmt3b*-WT mice and deficient (KO) mice with or without thioacetamide (TAA) treatment. Scale bar, 100 µm. (**C**) The proportion of 8-OHdG-positive hepatocytes in each genotype. The number of 8-OHdG-positive hepatocytes and the gross number of hepatocytes were counted in five random fields of view (400 × magnification). The average rate of 8-OHdG-positive hepatocytes is represented as the percentage. P values were determined using the two-tailed *t* test. Data are presented as the mean ± s.d. (**D**) Fluorescence percentage of control primary hepatocytes (*Dnmt3b* WT). A set of control primary hepatocytes and *Dnmt3b*-KO primary hepatocytes were cultured simultaneously; the fluorescence level, indicating cellular ROS activity, was measured after the cells were stained with 2′,7′-dichlorofluorescin diacetate (DCFDA). The difference between the genotypes were determined as the percentage of the control after background subtraction. The P value was determined using the one-sample *t* test. Data are presented as the mean ± s.d. Images in (**A**), (**C**) and (**D**) were made on GraphPad Prism ver 7.00.

Given that many components of mitochondrial respiratory complexes are downregulated in the absence of *Dnmt3b*, we hypothesized that *Dnmt3b* deficiency interrupts mitochondrial respiration in hepatocytes, and the accumulated oxidative stress could enhance inflammation, resulting in carcinogenesis. To test this theory, we measured mitochondrial respiration in primary hepatocytes collected from the *Dnmt3b*-WT and deficient mice. Although the oxygen consumption rate (OCR) for basal respiration or ATP-linked respiration was not suppressed, the spare respiratory capacity significantly decreased in response to treatment with the mitochondrial uncoupler p-trifluoromethoxy carbonyl cyanide phenyl hydrazine (FCCP) in *Dnmt3b*-deficient hepatocytes (Fig. 6A).

To examine the oxidative stress resulting from mitochondrial dysfunction, we measured the level of reactive oxygen species (ROS) in the *Dnmt3b*-deficient liver. We performed immunohistochemical staining of 8-hydroxy-2′-deoxyguanosine (8-OHdG), which indicates the level of DNA damage caused by oxidative stress. The *Dnmt3b*-deficient mice exhibited a higher number of hepatocytes with a stronger nucleus staining than the control mice under both TAA-free and TAA-treated conditions (Fig. 6B). Under TAA treatment, the proportion of 8-OHdG-positive cells in five random fields of view were significantly higher in the *Dnmt3b*-deficient liver (Fig. 6C). To quantify the ROS level in viable cells, we cultured primary hepatocytes from each genotype and measured the 2′,7′-dichlorofluorescin diacetate (DCFDA) fluorescence level. We observed that the primary hepatocytes derived from the *Dnmt3b*-deficient mice showed a higher ROS level than those from the WT mice (Fig. 6D).

These data collectively indicate that *Dnmt3b* deletion reduced the mitochondrial respiratory capacity under stress, leading to increased oxidative stress, which could be closely associated with enhanced hepatitis, fibrosis and hepatocarcinogenesis.

## Discussion

Dysregulation of DNA methylation is commonly observed in various malignant tumors. In the current study, we first demonstrated that DNMT3B was highly expressed in the HCC tissue than in the non-tumorous liver tissue but majority was the isoforms lacking catalytic activity, consistent with a previous report^19,20,26^. We noticed that exon 21, one of the exons corresponding to catalytic domain, was skipped in considerable rate of DNMT3B mRNAs expressed in cirrhotic liver RNs. These data indicated that dysregulation of DNMT3B could be triggered during the course of inflammation-associated hepatocarcinogenesis. On the other hand, chronic hepatitis tissues showed a comparable level of exon 21-skipping rate to normal liver tissues. We speculate that it could be because cirrhotic liver RNs consist of clonal population of hepatocytes under chronic inflammation although further studies for DNMT3B isoform expressed in inflamed liver tissues will be needed.

We explored the role of *Dnmt3b* on chronic hepatitis and hepatocarcinogenesis using a novel *Dnmt3b*-deficient mouse model through deletion of exons 15–19, including the PC motif (exon 18) and ENV motif (exon 19), which are essential for methylation activity. We revealed that the loss of *Dnmt3b* exacerbated hepatitis and promoted the progression of fibrosis, cirrhosis, and carcinogenesis in the inflamed liver; however, *Dnmt3b* deletion exhibited less effect on liver tissues without inflammation. The presence of several Ki67-positive cells in the inflammatory liver tissue in *Dnmt3b*-deficient mice suggested that the tissue regeneration and cell proliferation was promoted by the exacerbation of hepatitis. The *Dnmt3b*-deficient mice exhibited progression of liver fibrosis upon long-term treatment with TAA, compared to the control mice; these characteristics resembled the pathological features of human liver cirrhosis.

As expected, the methylation level at the CpG sites of the genome was decreased in the liver of the *Dnmt3b*-deficient mice compared to that in the control mice, as observed using the WGBS technique; however, the difference between these values was not considerable. A possible explanation for this result could be that another de novo DNMT, Dnmt3a, could have compensated for the loss of Dnmt3b^31^. Although the reduction in the methylation level was similar across the genome, most DMRs were located at the gene body. These data are consistent with a previous report showing that Dnmt3b can induce methylation at cytosines located at the gene body^22^. The functional annotation analysis revealed that many genes bearing DMRs were classified into the cluster of transcription regulation. These genes include *Sox9, Sall1* and *Mdk,* all of which may relate to cell pluripotency or hepatocarcinogenesis^32–34^. Notably, the length of the DMR residing on a gene body showed a positive correlation with changes in gene expression. Although the correlation was weak, these results suggest that Dnmt3b is the main molecule to construct gene body methylation, which can also alter gene expression levels through a mechanism different from promoter methylation, such as the elevation of spurious transcripts starting at the gene body.

GSEA analysis derived from RNAseq data revealed that the expression of genes associated with oxidative phosphorylation was decreased in the liver tissues of the *Dnmt3b*-deficient mice, regardless of TAA treatment. Oxidative phosphorylation is a metabolic pathway that produces ATP using enzymes that oxidize nutrients in the mitochondria. Inactivation of oxidative phosphorylation caused by mitochondrial dysfunction decreases ATP synthesis and induces excessive ROS production. In human chronic hepatitis, such as hepatitis B, hepatitis C, and non-alcoholic steatohepatitis, persistent inflammatory stimulation causes ROS production via the NF-κB pathway^35–38^. Exposure to ROS can cause genomic alterations and contribute to cancer development^39^. We demonstrated that a lot of genes encoding mitochondrial components were downregulated in the *Dnmt3b*-deficient mice. Primary hepatocytes isolated from the *Dnmt3b*-deficient mice exhibited a reduction in the mitochondrial spare respiratory capacity. These results are consistent with a recent report from Cieslar-Pobuda et al. that suggests that DNMT3B is important for balanced mitochondrial biogenesis and respiration in human embryonic stem cells^40^. The present findings indicate that TAA treatment increased mitochondrial dysfunction and ROS production in the hepatocytes of *Dnmt3b*-deficient mice, leading to the exacerbation of hepatitis. A possible cause of the inactivation of oxidative phosphorylation could be that the methylation level at the gene body of *Sirt4* was decreased in the *Dnmt3b*-deficient liver. Sirt4 mainly localizes in the mitochondria^41^ and regulates mitochondrial function in the liver^30^. In our transcriptome analysis, the expression level of *Sirt4* was elevated in the *Dnmt3b*-deficient mice; however, it was not significantly different from control mice. *Ppara* and *Ppargc1a*, which regulate mitochondrial fatty acid oxidation and lipid droplet formation, had short DMRs less than 100 bps in the gene bodies, but expression levels of these genes did not significantly differ between *Dnmt3b*-WT and *Dnmt3b*-deficient livers. As a large level of the lipid accumulation was observed in aged livers both from *Dnmt3b*-WT and *Dnmt3b*-deficient mice, future studies that compare hepatic steatosis distributions patterns in younger mice may aid in elucidating the mechanisms behind the mitochondrial dysfunction that occur under *Dnmt3b* loss.

In summary, using a novel mouse model, we demonstrated that *Dnmt3b* depletion in hepatocytes exacerbated inflammation in chronic hepatitis leading to accelerated fibrosis and carcinogenesis. Loss of *Dnmt3b* resulted in a decrease in the methylation level in the genome, mainly at the gene body, and mitochondrial dysfunction through inactivation of oxidative phosphorylation. Our findings indicate that *Dnmt3b* plays an important role in protecting hepatocytes in chronic hepatitis. Further examination of *Dnmt3b* function is necessary to clarify the molecular basis of DNA methylation with respect to inflammation-associated hepatocarcinogenesis.

## Materials and methods

### Patients and sample collection

Of 247 patients who underwent hepatectomy between January 2009 and June 2019 at Kyoto University Hospital, 17 patients with HCC were selected and subjected to RNAseq. The study protocols were approved by the ethics committee of Kyoto University. HCC samples were obtained with written informed consent or based on an opt-out method of consent. Informed consent was obtained based on the Study Protocol G1084, and opt-out method of consent was obtained based on the Study Protocol G616, which was linked to G1084 protocol. For control groups, seven RNAseq datasets of normal liver tissue provided by living donors for liver transplantation, and fifteen RNAseq datasets of RN tissues provided by patients who underwent living-donor liver transplantation with a diagnosis of liver cirrhosis and decompensated liver failure^42^, were obtained from National Bioscience Database Center database (Research ID, hum0138; Japanese Genotype-phenotype Archive Data set ID, JGAD000203). In addition, forty RNAseq datasets of HCV-positive non-cancerous chronic hepatitis tissues were obtained from the Japanese cohort of International Cancer Genome Consortium (ICGC) database (Project code, LIRI-JP). The details are described in Supplementary Materials and Methods. The downloaded data were analyzed on the same pipeline as that used for our hospital's data on HCC. This research conformed to the provisions of the Declaration of Helsinki.

### Calculation of exon-skipping rate

From the RNAseq data mentioned above, the number of reads that proved the inclusion (Ri) or skipping (Rs) of exons were obtained. Reads that spanned introns were excluded. The rate of an mRNA skipping the exon was estimated as follows:

$$ {\text{Exon}}\;{\text{ skipping}}\;{\text{ rate }} = \frac{{{\text{Rs}}}}{{{\text{Ri}} + {\text{Rs}}}} $$

Samples that did not have any reads around the exon were excluded from analysis.

### Mice

For generating *Dnmt3b*-floxed mice, a targeting vector was designed such that the exon 15–19 sequence of *Dnmt3b* was flanked by two *loxP* sequences, which were located respectively inside the 5′ and 3′ homologous arms, with the *FRT*-flanked Neo cassette residing right after the first *loxP* site (Fig. 1A). Each fragment was created by PCR using a bacterial artificial chromosome clone as a template and sequentially subcloned into pBS-DTA (*Kpn*I). The targeting vector was injected into the pronuclei of C57BL/6 ES cells to generate the founder mice. The transgene-positive founder mice were crossed with *FLP* deleter mice to delete the Neo cassette.

The *ALB*-*Cre* mice^43^ were a gift from the Center for iPS Cell Research and Application, Kyoto University; they were maintained by self-crossing between heterozygous mice. All mice were maintained in a specific pathogen-free facility at the Kyoto University Faculty of Medicine.

Primers for genotyping PCR are listed in the Supplementary Materials and Methods.

### Animal experiments

TAA (Sigma-Aldrich, St. Louis, MO) was dissolved in drinking water at a concentration of 0.02%, starting at the age of 8–10 weeks. Animals were euthanized using carbon dioxide or anesthetized by injecting chloral hydrate peritoneally. When counting tumors, liver was sliced sagittally into 2 mm thick sections and examined. The tumor volume was calculated by using the formula: Tumor volume = Length × Width^2^/2. All animal experiments were approved by the Ethics Committee for Animal Experiments and performed under the Guidelines for Animal Experiments of Kyoto University.

### Histologic analyses

For histologic analyses, liver tissues fixed with 4% paraformaldehyde were embedded in paraffin. Paraffin-embedded tissues were sectioned and stained with hematoxylin and eosin or Masson’s trichrome.

### Immunohistochemical staining

For anti-Dnmt3b and anti-CD19 staining, target retrieval was performed in Target Retrieval Solution, pH 9 (Agilent, Santa Clara, CA); otherwise, it was performed in Target Retrieval Solution, Citrate, pH 6.1 (10 ×) (Agilent), following the manufacturer’s protocol. The sections were incubated with the primary antibodies and, subsequently, with ImmPRESS peroxidase-conjugated secondary antibodies (Vector Laboratories, Burlingame, CA) and visualized with the ImmPACT DAB Substrate Kit (Vector Laboratories). The slides were counterstained with hematoxylin (FUJIFILM Wako, Osaka, Japan). The primary antibodies used are presented in Supplementary Materials and Methods.

### Quantification of the fibrosis area

The fibrosis area was quantified on the images of liver stained with Masson’s trichrome using ImageJ (National Institute of Health, Bethesda, MD) after splitting the image based on the color channels.

### RNA extraction

Total RNA was extracted from 17 human HCC tissues, 15 human cirrhotic RN tissues, and liver tissues of *Dnmt3b*-WT and deficient mice (N = 4 for both genotypes not treated with TAA and N = 4 for both genotypes treated with TAA for 4 weeks) using the RNeasy Mini kit (QIAGEN, Hilden, Germany), following the manufacturer’s protocol.

### Quantitative real-time polymerase chain reaction (RT-qPCR)

The isolated RNA was reverse transcribed using ReverTra Ace qPCR RT Master Mix (Toyobo, Osaka, Japan). Quantitative real-time PCR was performed with LightCycler 480 System II (Roche, Basel, Switzerland) using the primers listed in Supplementary Materials and Methods. Each sample was measured in duplicate. All data were normalized to the expression level of 18 s ribosomal RNA, measured using a TaqMan probe, 5′-CGCCTGGATACCGCAGCTAGGAATAATG-3′.

### Whole-genome bisulfite sequencing (WGBS)

DNA was isolated from mouse liver tissues (N = 1 for both genotypes) using DNeasy Blood & Tissue Kit (QIAGEN). WGBS library preparation, sequencing, mapping, and methylation analysis were performed by Novogene (Beijing, China). The details are described in Supplementary Materials and Methods. Functional annotation analysis was conducted with DAVID Bioinformatics Resources 6.8^44^.

### Bisulfite sequencing

Bisulfite conversion of genomic DNA was performed using EpiTect Bisulfite Kit (QIAGEN). Primers were designed using MethPrimer^45^ to cover a specific DMR as much as possible, as shown in the Supplementary Materials and Methods. PCR was performed using EpiTaq HS (Takara Bio) according to the manufacturer’s instructions. Targeted deep sequencing on the amplified fragments of DMRs with multiplexed tags were conducted using the IonProton sequencer (Thermo Fischer Scientific, Waltham, MA) according to the manufacturer’s protocol. Sequence reads were aligned using Bismark Bisulfite Mapper v0.21.0^46^ on mouse reference genome mm10. The methylation rate at each CpG site was calculated by considering the frequency of C>T transitions at each locus after a bisulfite conversion.

### RNA sequencing (RNAseq)

RNAseq was performed using the Novaseq 6000 (Illumina, San Diego, CA) platform. The details are described in Supplementary Materials and Methods.

### Primary hepatocyte isolation

Primary hepatocytes were isolated from mice, aged 6–8 weeks, under anesthesia by intraperitoneal injection of chloral hydrate. The details are described in Supplementary Materials and Methods.

### Measurement of oxygen consumption rate (OCR) of cells

On a Seahorse XF96 Cell Culture Microplate (Agilent) coated with Collagen I (Corning #354236, Corning, NY), 20,000 primary hepatocytes were seeded per well in DMEM + 10% FBS + Pen/Strep. The OCRs were measured on a Seahorse XF analyzer using the XF Cell Mito Stress Test (Agilent). Briefly, the compounds, oligomycin, FCCP and a mixture of rotenone and antimycin, were serially injected at each indicated timepoint to measure ATP-linked respiration, maximal respiration, and non-mitochondrial respiration, respectively. Five replicates were measured from each mouse.

### Measurement of reactive oxygen species (ROS) activity within cells

On a 96-well plate coated with Collagen I, 40,000 primary hepatocytes were seeded per well in DMEM + 10% FBS + Pen/Strep. The ROS activity level within the primary hepatocytes was measured using the DCFDA/H2DCFDA—Cellular ROS Assay Kit (ab113851, Abcam, Cambridge, UK) following the manufacturer’s protocol. Briefly, the cells were washed once with the buffer included in the kit and stained with 25 µM DCFDA for 45 min at 37 °C. After washing once with the buffer, the fluorescence levels were measured at Ex/Em = 485/535 nm. Five replicates were measured from each mouse.

### Statistical analysis

Statistical analysis was performed using Student’s *t* test or Tukey’s multiple comparison test for differences between two groups or multiple groups, respectively. *P* < 0.05 was considered significant. All tests were performed in GraphPad Prism ver 7.00 (https://www.graphpad.com GraphPad Software, La Jolla, CA).

## Supplementary information

Supplementary Tables.

Supplementary Information 1.

## Data Availability

Patients’ sequence datasets are available in the Japanese Genotype-phenotype Archive (JGA, http://trace.ddbj.nig.ac.jp/jga), which is hosted by the DDBJ, under accession number JGAS000234. Mice sequencing datasets are available at the DDBJ Sequence Read Archive (DRA, https://www.ddbj.nig.ac.jp/dra/index-e.html) under accession number DRA010641.

## Abbreviations

8-OHdG: 8-Hydroxy-2′-deoxyguanosine
DCFDA: 2′,7′-Dichlorofluorescin diacetate
DMR: Differentially-methylated region
DNMT: DNA methyltransferase
FCCP: P-Trifluoromethoxy carbonyl cyanide phenyl hydrazine
FPKM: Fragments per kilobase million
GSEA: Gene set enrichment analysis
HCC: Hepatocellular carcinoma
NES: Normalized enrichment score
OCR: Oxygen consumption rate
RN: Regenerative nodule
RNAseq: RNA sequencing
ROS: Reactive oxygen species
RT-qPCR: Quantitative real-time polymerase chain reaction
TAA: Thioacetamide
WGBS: Whole-genome bisulfite sequencing
