# Author Correction: DNA methyltransferase 3B plays a protective role against hepatocarcinogenesis caused by chronic inflammation via maintaining mitochondrial homeostasis

**DOI:** 10.1038/s41598-021-93371-w

**Published:** 2021-07-01

**Authors:** Eriko Iguchi, Atsushi Takai, Haruhiko Takeda, Ken Kumagai, Soichi Arasawa, Yuji Eso, Takahiro Shimizu, Yoshihide Ueda, Hiroyuki Marusawa, Hiroshi Seno

**Affiliations:** 1 Department of Gastroenterology and Hepatology, Graduate School of Medicine, Kyoto University, 54 Kawahara-cho, Shogoin, Sakyo-ku, Kyoto, 606-8507 Japan; 2 Department of Gastroenterology and Hepatology, Graduate School of Medicine, Kobe University, Hyogo, Japan; 3 Department of Gastroenterology and Hepatology, Osaka Red Cross Hospital, Osaka, Japan

Correction to: *Scientific Reports* https://doi.org/10.1038/s41598-020-78151-2, published online 04 December 2020

The original version of this Article contained an error in the accession number in the Data Availability section, where

“Patients’ sequence datasets are available in the Japanese Genotype–phenotype Archive (JGA, http://trace.ddbj.nig.ac.jp/jga), which is hosted by the DDBJ, under accession number JGAS00000134.”

now reads:

“Patients’ sequence datasets are available in the Japanese Genotype-phenotype Archive (JGA, http://trace.ddbj.nig.ac.jp/jga), which is hosted by the DDBJ, under accession number JGAS000234.”

Additionally, the Patients and sample collection section in the original version of this Article was incorrect.

“Of 247 patients who underwent hepatectomy between January 2009 and June 2019 at Kyoto University Hospital, 17 patients with HCC were selected and subjected to RNAseq. Out of 34 patients who underwent living-donor liver transplantation with a diagnosis of liver cirrhosis and decompensated liver failure between January 2014 and October 2015, nine cases were randomly selected and 15 RN tissues were manually collected from the explanted liver sections. For control samples, we used existing RNAseq data of seven normal liver tissues provided by living donors for liver transplantation, which were collected in our previous study^42^. Written informed consent was obtained from all the patients and donors mentioned above. In addition, 40 RNAseq datasets of HCV-positive HCCs were obtained from the Japanese cohort of International Cancer Genome Consortium (ICGC) database (Project code, LIRI-JP). The details are described in Supplementary Materials and Methods. The downloaded data on chronic hepatitis were analyzed on the same pipeline as that used for our hospital's data on HCC, RN and normal liver samples. This research conformed to the provisions of the Declaration of Helsinki. The study protocol was approved by the ethics committee of Kyoto University.”

now reads:

“Of 247 patients who underwent hepatectomy between January 2009 and June 2019 at Kyoto University Hospital, 17 patients with HCC were selected and subjected to RNAseq. The study protocols were approved by the ethics committee of Kyoto University. HCC samples were obtained with written informed consent or based on an opt-out method of consent. Informed consent was obtained based on the Study Protocol G1084, and opt-out method of consent was obtained based on the Study Protocol G616, which was linked to G1084 protocol. For control groups, seven RNAseq datasets of normal liver tissue provided by living donors for liver transplantation, and fifteen RNAseq datasets of RN tissues provided by patients who underwent living-donor liver transplantation with a diagnosis of liver cirrhosis and decompensated liver failure^42^, were obtained from National Bioscience Database Center database (Research ID, hum0138; Japanese Genotype-phenotype Archive Data set ID, JGAD000203). In addition, forty RNAseq datasets of HCV-positive non-cancerous chronic hepatitis tissues were obtained from the Japanese cohort of International Cancer Genome Consortium (ICGC) database (Project code, LIRI-JP). The details are described in Supplementary Materials and Methods. The downloaded data were analyzed on the same pipeline as that used for our hospital's data on HCC. This research conformed to the provisions of the Declaration of Helsinki."

The original Article and accompanying Supplementary Information file have been corrected.

