# Supplementary material for: DNA methyltransferase 3B plays a protective role against hepatocarcinogenesis caused by chronic inflammation via maintaining mitochondrial homeostasis
Source: Sci Rep. 2020 Dec 4;10:21268. doi: 10.1038/s41598-020-78151-2 (PMC7719166; doi:10.1038/s41598-020-78151-2)
Supplement: Supplementary file 2 — Supplementary Information 1. [file 41598_2020_78151_MOESM2_ESM.pdf]

Supplementary Information

DNA methyltransferase 3B plays a protective role against hepatocarcinogenesis caused by chronic inflammation via maintaining mitochondrial homeostasis

Eriko Iguchi[1], Atsushi Takai[1], Haruhiko Takeda[1], Ken Kumagai[1], Soichi Arasawa[1], Yuji Eso[1], Takahiro Shimizu[1], Yoshihide Ueda[2], Hiroyuki Marusawa[3] and Hiroshi Seno[1]

[1] Department of Gastroenterology and Hepatology, Graduate School of Medicine, Kyoto University, Kyoto, Japan

[2] Department of Gastroenterology and Hepatology, Graduate School of Medicine, Kobe University, Hyogo, Japan

[3] Department of Gastroenterology and Hepatology, Osaka Red Cross Hospital, Osaka, Japan

Supplementary Figure S1

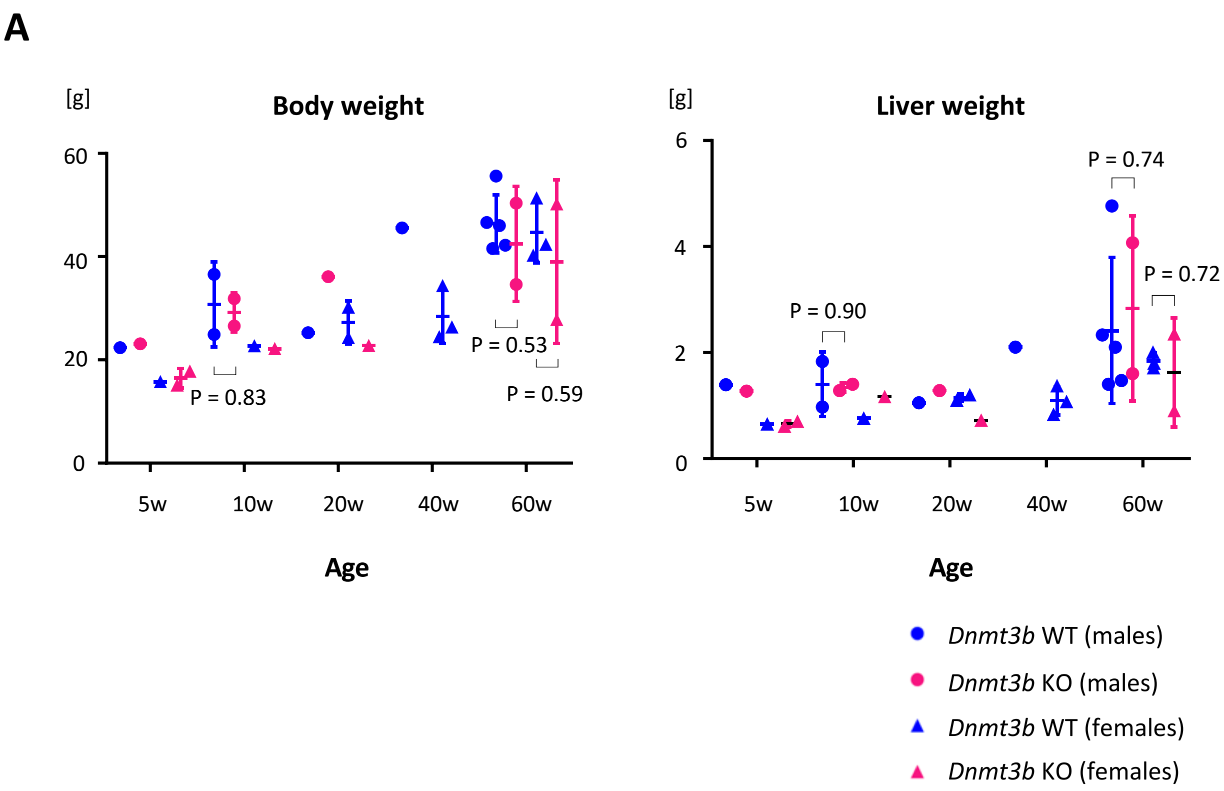

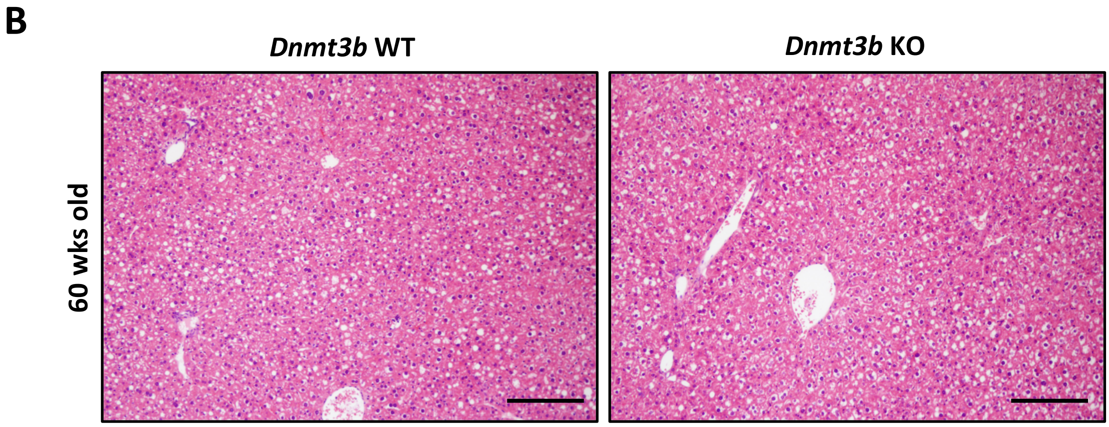

Supplementary Figure S2

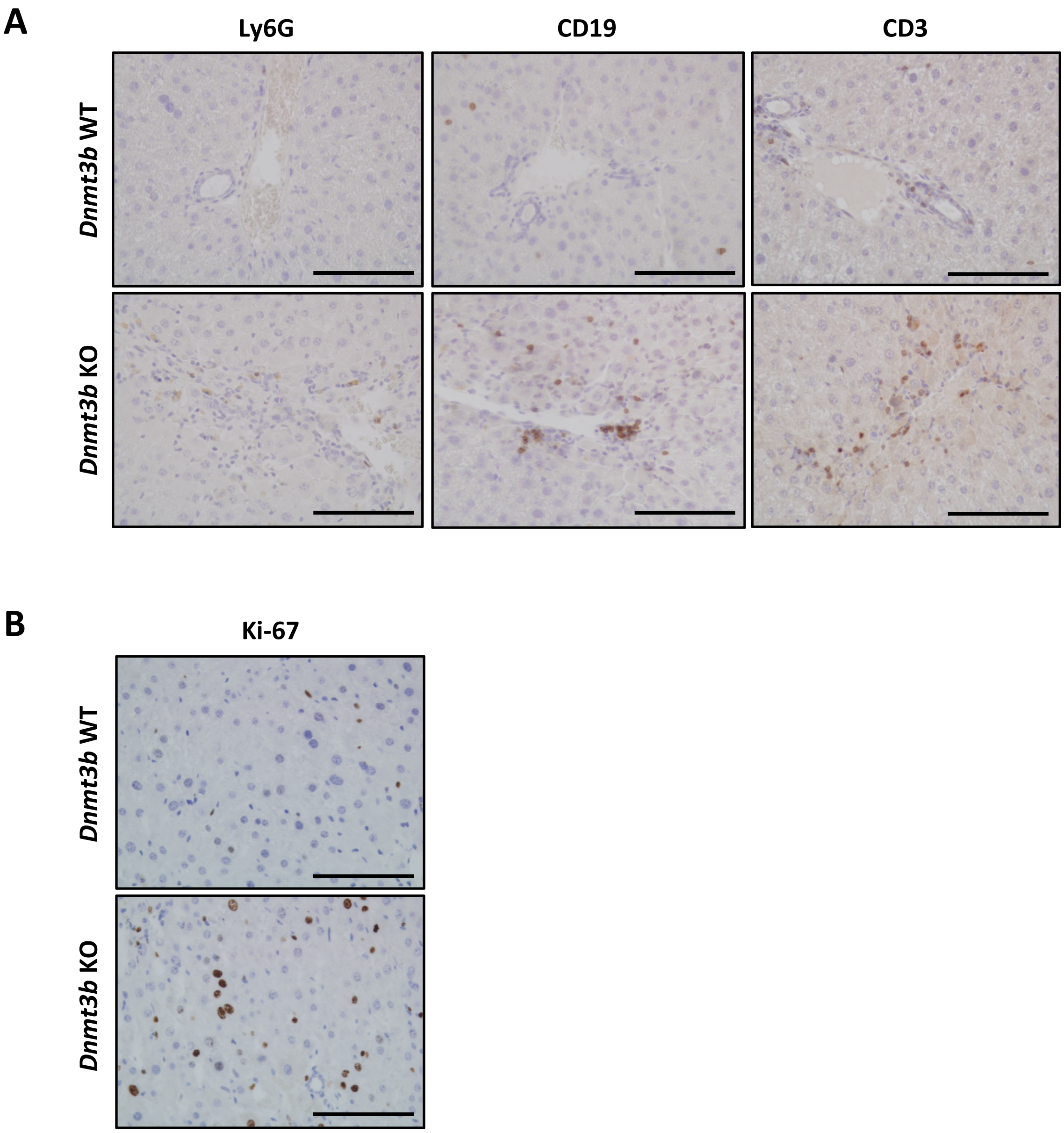

Supplementary Figure S3

A

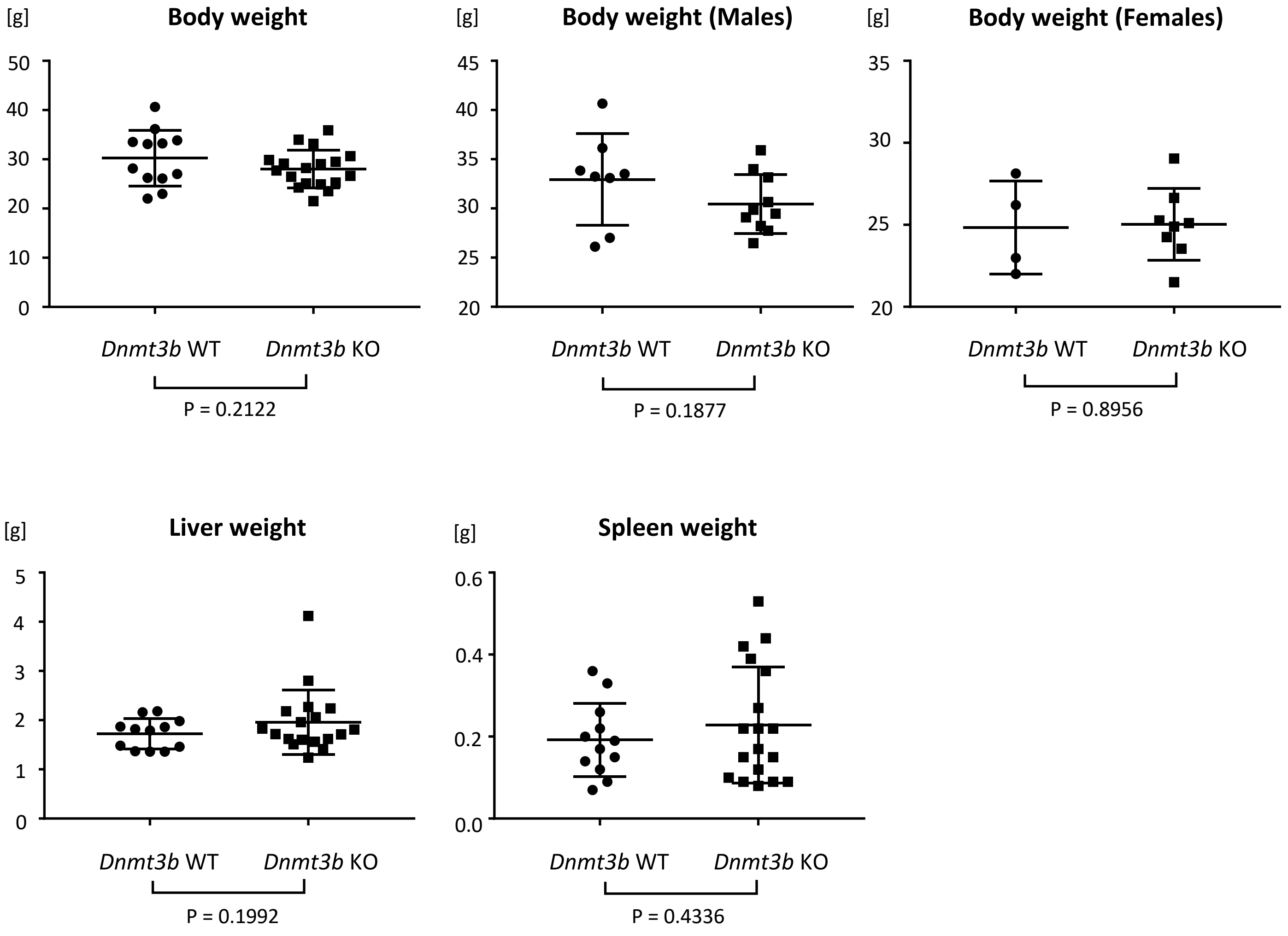

B

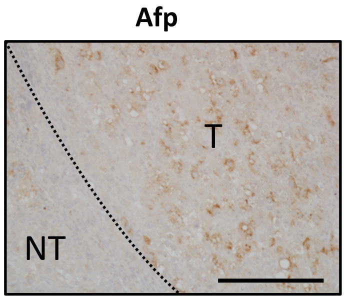

Supplementary Figure S4

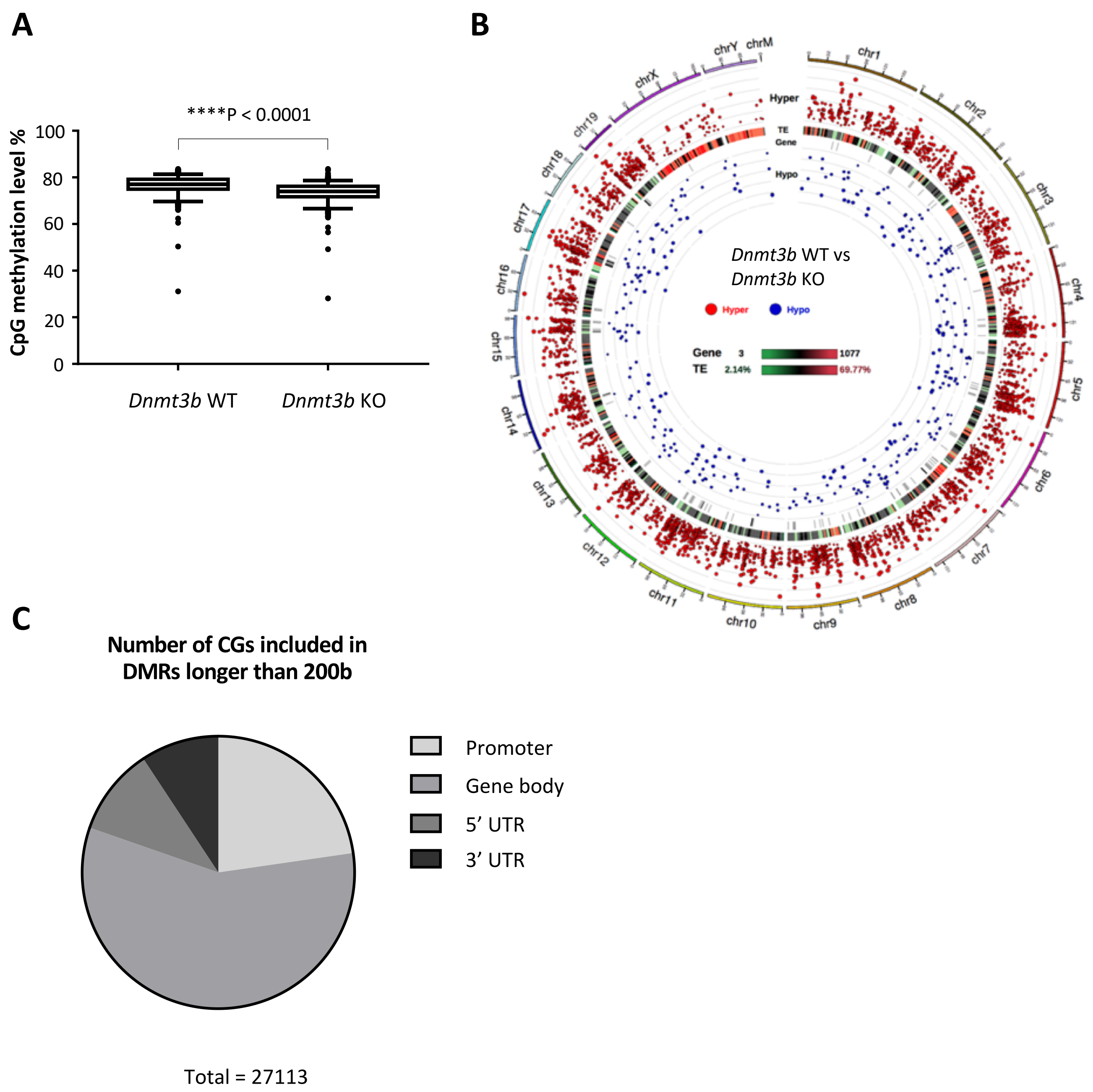

Supplementary Figure S5

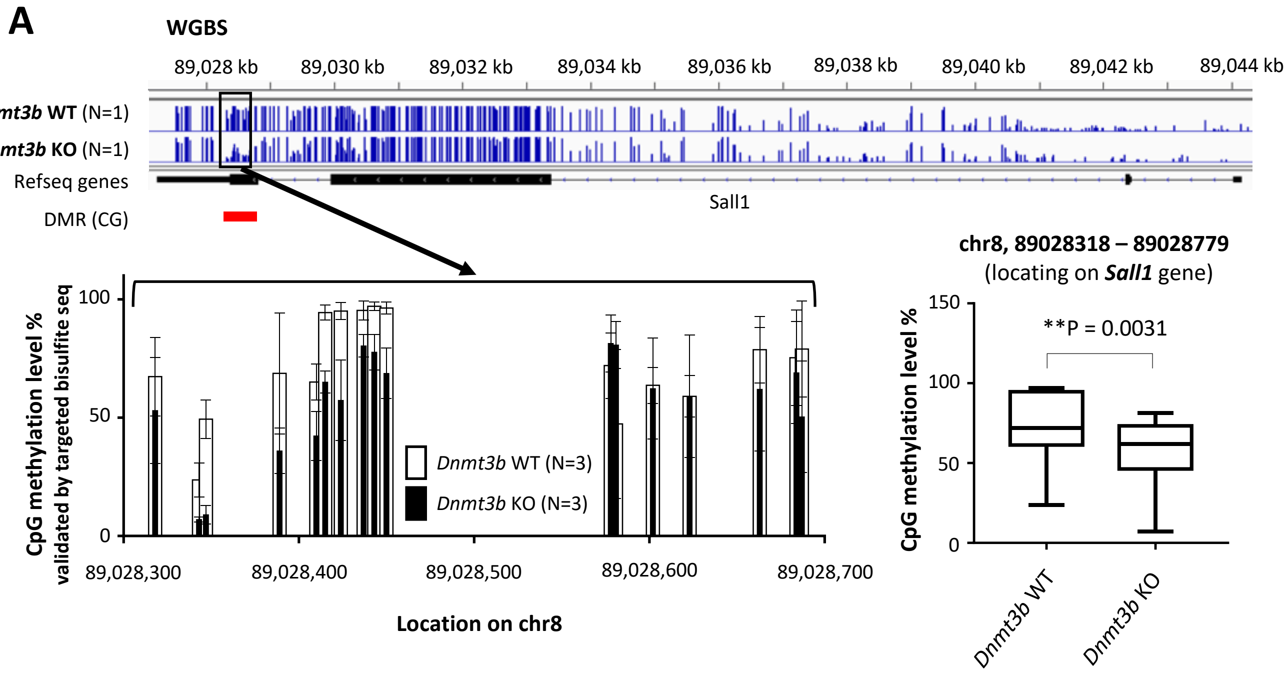

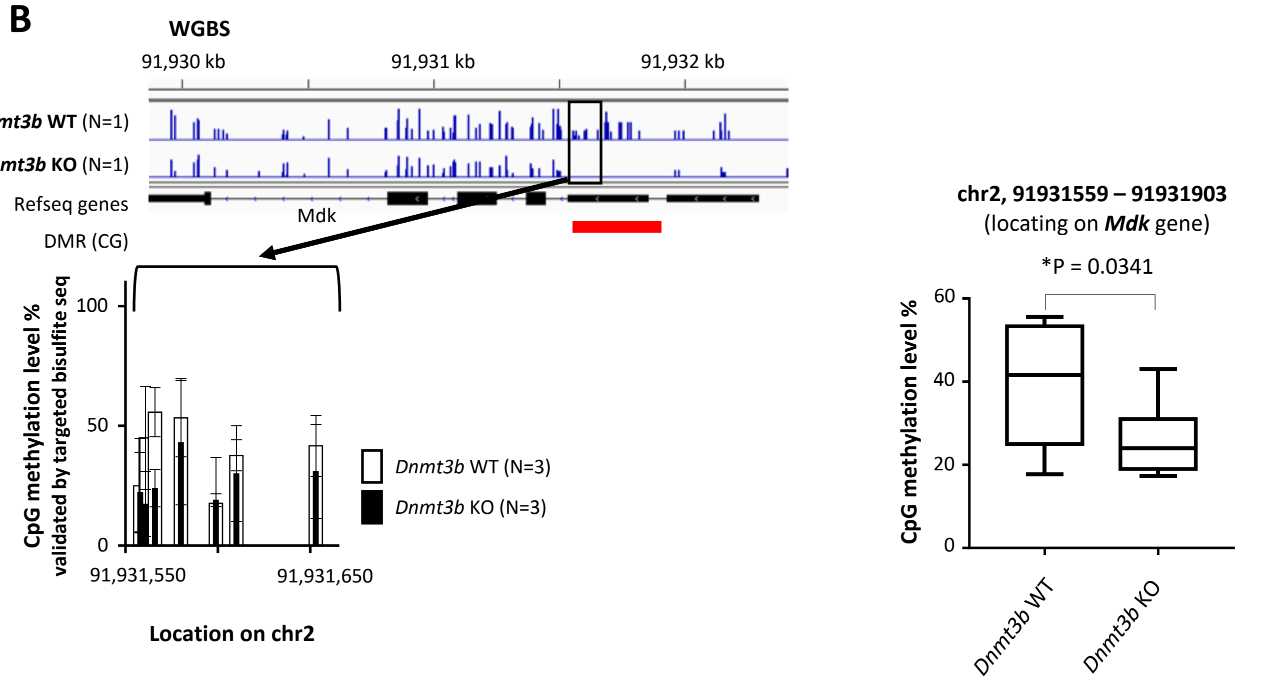

Supplementary Figure S6

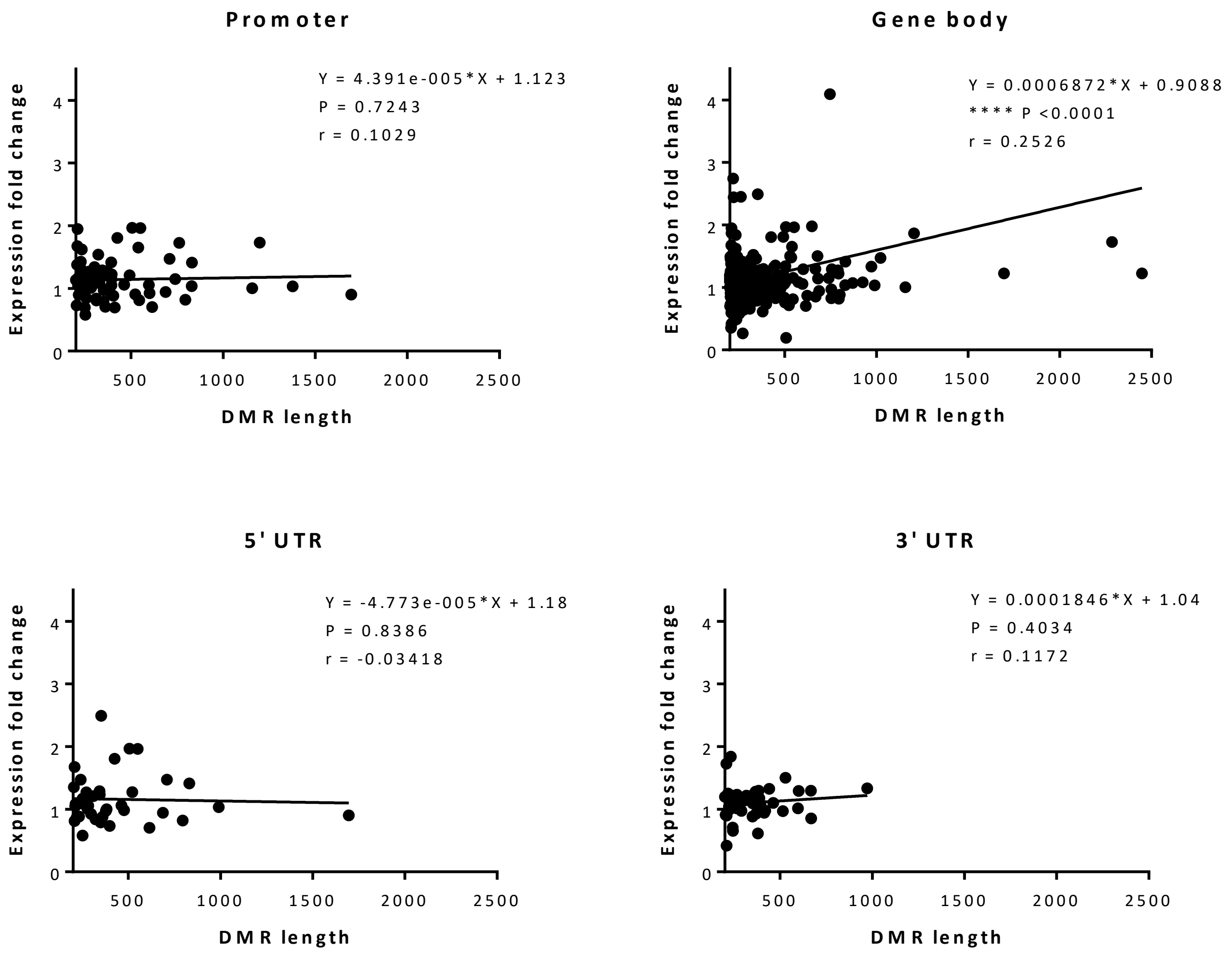

Supplementary Figure S7

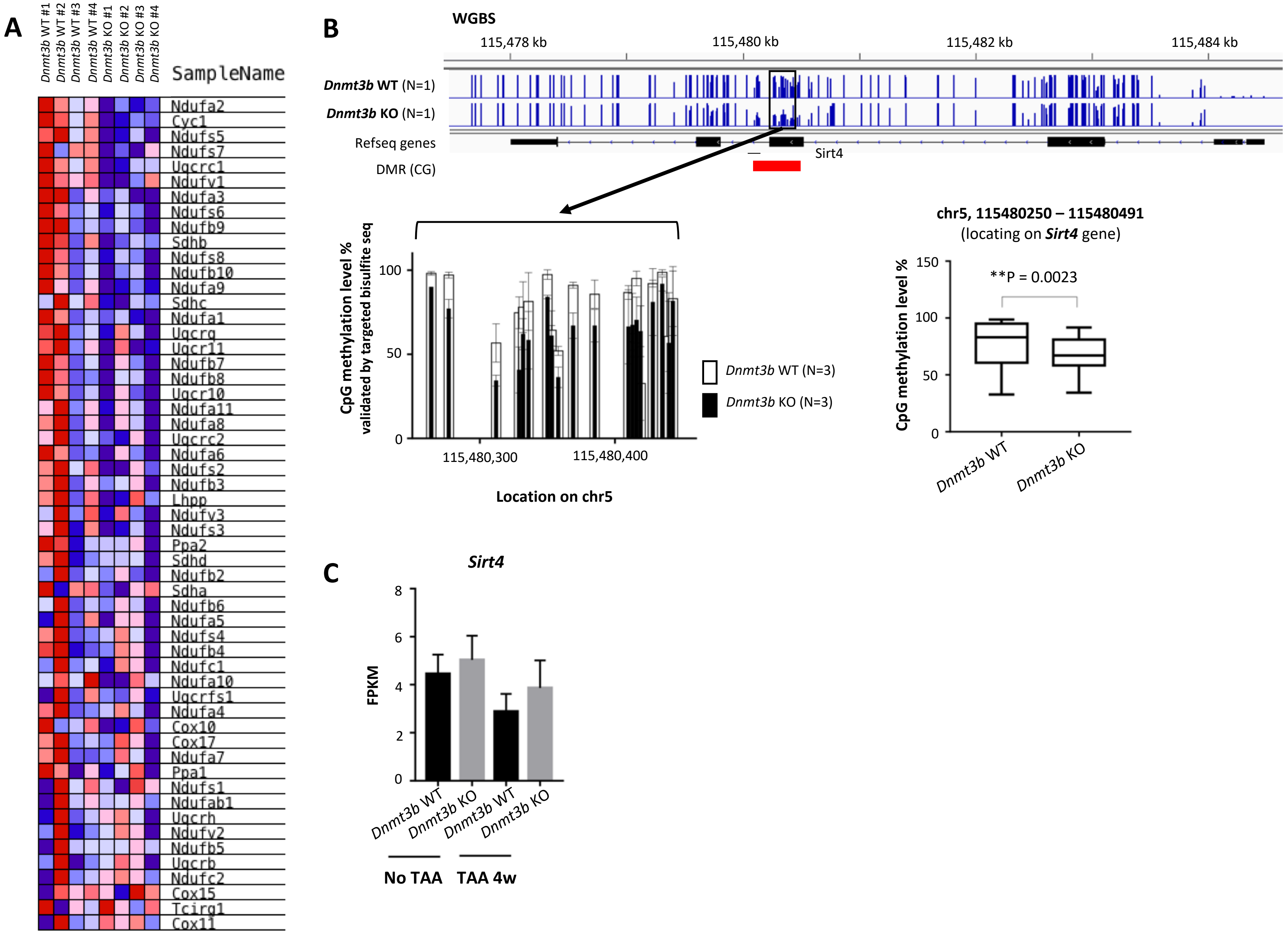

Supplementary Figures

Supplementary Figure S1. Hepatocyte-specific *Dnmt3b*-deficient (KO) mice had normal systemic development and liver growth.

(A) Body weight and liver weight of *Dnmt3b*-WT and KO mice at multiple time points during their growth. P value was determined by the multiple t test. Data are presented as the mean ± s.d. Images were made on GraphPad Prism ver 7.00. (B) Representative HE-stained images of liver sections from *Dnmt3b*-WT and KO mice at the age of 60 weeks. Scale bar, 200 µm.

Supplementary Figure S2. Thioacetamide (TAA)-induced hepatitis was enhanced in *Dnmt3b*-deficient (KO) mice.

Representative Ly6G, CD19, CD3 (A) and Ki-67 (B) immunohistochemical staining of liver sections from a *Dnmt3b*-WT mouse and KO mouse after 4 weeks of 0.02% TAA treatment. Scale bar, 100 µm.

Supplementary Figure S3. Hepatocyte-specific *Dnmt3b*-deficient (KO) mice had the same level of body and organ weight as control mice after 30-week thioacetamide (TAA) treatment.

(A) Body weight, liver weight, and spleen weight of *Dnmt3b*-WT and KO mice measured after 30 weeks of TAA treatment. P value is determined by the two-tailed t test. Data are presented as the mean ± s.d. Images were made on GraphPad Prism ver 7.00. (B) Representative image of *Afp* immunohistochemical staining in liver sections of *Dnmt3b*-KO mice. Scale bar, 200 µm.

Supplementary Figure S4. *Dnmt3b*-deficient (KO) liver showed lower CpG methylation level, forming differentially methylated regions.

(A) Distribution of CpG methylation level calculated with 10 kb as a bin in each sample. Whiskers show 5-95 percentile. P value is determined by the two-tailed paired t test. (B) CG-context DMR circos plot is shown. The first layer: the red dots indicate significantly hypermethylated regions in the *Dnmt3b*-WT liver. The second layer: TE, repeat original proportion of the heat map. The third layer: heat density map of the gene. The fourth layer: the blue dots indicate significantly hypomethylated regions in the *Dnmt3b*-WT liver. (C)

Distribution of CpGs included in DMRs longer than 200b. Images in (A) and (C) were made on GraphPad Prism ver 7.00.

Supplementary Figure S5. Validation of WGBS results by targeted bisulfite sequencing.

(top) CpG methylation level in *Sall1* (A) or *Mdk* (B) gene obtained from WGBS. DMR is indicated by a red rectangle. (bottom, left) CpG methylation level at each CpG site in the DMR calculated by targeted bisulfite sequencing, conducted on the loci where primers could be designed, shown by black rectangles. Data are presented as the mean ± s.d. N=3 for both genotypes. (bottom, right) The overall methylation level in this DMR. Whiskers show from minimum to maximum. P value is determined by the two-tailed paired t test. Images were made on GraphPad Prism ver 7.00.

Supplementary Figure S6. Correlation between methylation change in each gene functional element and gene expression change.

Correlation between the total length of all DMRs locating on one gene functional element and the fold change of the gene expression level. Gene expression level was obtained from FPKM values in RNA-seq data (N=4 for both genotypes). Images were made on GraphPad Prism ver 7.00.

Supplementary Figure S7. *Nduf* genes were downregulated accompanied with *Sirt4* upregulation in *Dnmt3b*-deficient (KO) liver.

(A) Heatmap illustrating the expression level of genes included in the gene set KEGG_OXIDATIVE_PHOSPHORYLATION in thioacetamide (TAA)-treated mice. (B) (top) CpG methylation level in *Sirt4* gene obtained from WGBS. DMR is indicated by a red rectangle. (bottom, left) CpG methylation level at each CpG site in the DMR calculated by targeted bisulfite sequencing, conducted on the loci where primers could be designed, shown by black rectangles. Data are presented as the mean ± s.d. N=3 for both genotypes. (bottom, right) The overall methylation level in this DMR. Whiskers show from minimum to maximum. P value is determined by the two-tailed paired t test. (C) mRNA expression level of *Sirt4* obtained from

RNAseq. Data are presented as the mean ± s.d. Images in (B) and (C) were made on GraphPad Prism ver 7.00.

Supplementary Table S1.

List of differentially methylated regions (DMRs) in the CG context spanning longer than 200 bps that exhibit a lower mCG level in the *Dnmt3b*-deficient liver.

Supplementary Table S2.

List of 424 genes bearing CG-context differentially methylated regions (DMRs) longer than 200 bps with a lower methylation level in the *Dnmt3b*-deficient liver.

Supplementary Table S3.

Annotation clusters with enrichment score >1.4 are revealed through DAVID functional annotation analysis of the genes listed in Supplementary Table 2. Annotation cluster 1 (PCDHG-related) and 2 (UGT1A-related) can be dismissed because the majority in both gene sets are clustered on the genome, causing apparent enrichment by a single differentially methylated region (DMR).

Supplementary Table S4.

Gene sets significantly enriched in *Dnmt3b*-WT mice (upper table) and deficient mice (lower table) that were not subjected to thioacetamide (TAA) treatment. In these mice, no gene set was detected to be significantly enriched. Significant enrichment was defined as FDR Q value <0.01 and FWER P value <0.05.

Supplementary Table S5.

Gene sets significantly enriched in *Dnmt3b*-WT mice (upper table) and deficient mice (lower table) treated with thioacetamide (TAA) for four weeks. Significant enrichment was defined as FDR Q value <0.01 and FWER P value <0.05.

Supplementary Materials and Methods

Reanalysis of public database

RNAseq data of normal liver tissues and RN tissues were downloaded from the Japanese Genotype-phenotype Archive (JGA, http://trace.ddbj.nig.ac.jp/jga), which is hosted by the DDBJ, under accession number JGAS000134. To compare the transcriptomic profiles of chronic hepatitis tissues with those of normal liver, RN or HCC, we reanalyzed the datasets of Japanese patients with HCV-related HCC in the International Cancer Genome Consortium (ICGC) dataset. RNAseq datasets of 40 non-tumor tissues from HCV-related HCC patients randomly selected were downloaded from the ICGC Data Portal (https://dcc.icgc.org/). FASTQ files of each dataset were processed and analyzed by the same protocol as our dataset.

Primers used for genotyping

	Forward	Reverse
Dnmt3b-floxed	5’-GGCTTTCCTTGATCTCTTACTCTTT-3’	5’-ACAACTAACTGGAGGTTCAAGACAG-3’
Cre	5'-AAAATTTGCCTGCATTACCG-3'	5'-ATTCTCCCACCGTCAGTACG-3'

Primers used for bisulfite-sequencing PCR

	Forward	Reverse
*Sox9*_1	5’- TTTTTAGGGGGTTTTTTTGGTATAT-3’	5’- AATCCAACTCAAAATTCAAAATTTAAA-3’
*Sox9*_2	5’- ATTTTTGGGATTTTAGATTTTTTTT-3’	5’- TACAAAACCCCTACTCTTAAAAACC-3’
*Sox9*_3	5’- GTTTTTAGGGGGTTTTTAAGAGTAG-3’	5’- TCAAATCAACTTTACCAACTTACAC-3’
Sirt4	5’- TTGTAGGATGGGTAAAGGTAATTGT-3’	5’- AAACCCTAAACCCCAACTAAAAC-3’
*Sall1*_1	5’- AGTTTTAGGGGTTAGAAGGTAAAGG-3’	5’- TCCTCTAACTAACCTAAAAAAAATAAC-3’
Sall1_2	5’- AGGTGAGTTGTTTTTATTGTTTA	5’- TCCTATCAAATTCCCAAAAATATT

	GGTT-3’	C-3’
Mdk	5’- GGATTTTTTATTTTTGATTGATT TGTT-3’	5’- ATAACTATAACCCCAATCCCTTCA-3’

Primers used for RT-qPCR

	Forward	Reverse
Dnmt3b	5’-GCGACAACCGTCCATTCTTC-3’	5’-TGAGCAGCAGACACCTTGATG-3’
Dnmt3a	5’-CTCCATAAAGCAGGGCAAAG-3’	5’-AGTCTCTGCCTCGCCAAG-3’
Il1b	5’-GCAACTGTTCCTGAACTCAACT-3’	5’-ATCTTTTGGGGTCCGTCAACT-3’
Tnfa	5’-GACGTGGAACTGGCAGAAGAG-3’	5’-CGATCACCCCGAAGTTCAGTAG-3’
Tbx3	5’-CCACCTCCAACAACACGTTCT-3’	5’-TAAGGAAACAGGCTCCCGAA-3’
Foxp1	5’-CCTCGCTCAAGGCATGATTC-3’	5’-AGGACTTGGAAGGTGCCGAG-3’
Sall1	5’-CAATCTGAAGGTCCACATGGGCAC-3’	5’-TGCCTCCTAGAAATGTCATGGG-3’
Mdk	5’-GTCAATCACGCCTGTCCTCT-3’	5’-CAAGTATCAGGGTGGGGAGA-3’
Ndufb8	5’-GGAATCGTGTGGACACGTCA-3’	5’-GTACTGCTTCGGACCCACAG-3’
Ndufa7	5’-CCCAGTCACAAGCTGTCCAA-3’	5’-TTGAGGGAGGCACAACTTCC-3’
Ndufa8	5’-CCCAGACAACAGACGACGAA-3’	5’-AGGAAAGAACACGAGATCGGC-3’
18s rRNA	5’-TAGAGTGTTCAAAGCAGGCCC-3’	5’-CCAACAAAATAGAACCGCGGT-3’

Antibodies used for immunohistochemical staining

	Host	Dilution	Manufacturer	Product #
Anti-Dnmt3b	rabbit	1:1000	NOVUS, Centennial, CO	NB300-516
Anti–Ki-67	rabbit	1:400	Cell Signaling Technology, Danvers, MA	#12202
Anti-F4/80	rat	1:500	Abcam, Cambridge, UK	ab6640
Anti-CD3	rabbit	1:100	Abcam, Cambridge, UK	ab16669
Anti-CD19	rabbit	1:800	Cell Signaling Technology, Danvers, MA	#90176S
Anti-Ly6G (Gr-1)	rat	1:500	Thermo Fisher Scientific, Waltham, MA	14-5931-82
Anti-8-OHdG	goat	1:1000	NOVUS, Centennial, CO	NB600-1508SS
Anti-Afp	goat	1:1000	Santa Cruz, Dallas, TX	sc-8108

Whole-genome bisulfite sequencing (WGBS)

A total of 5.2 μm of genomic DNA spiked with 26 ng of lambda DNA were fragmented by sonication to 200-300 bp with Covaris S220, followed by end repair and adenylation. Cytosine-methylated barcodes were ligated to sonicated DNA as per the manufacturer's instructions. Subsequently, these DNA fragments were treated twice with bisulfite using EZ DNA Methylation-GoldTM Kit (Zymo Research, Irvine, CA), before the resulting single-strand DNA fragments were subjected to PCR amplification using KAPA HiFi HotStart Uracil + ReadyMix (2×).

The library concentration was quantified by Qubit® 2.0 Flurometer (Life Technologies, Carlsbad, CA) and quantitative PCR; the insert size was assayed on Agilent Bioanalyzer 2100 system.

Bismark software (version 0.22.1) [46] was used to perform alignments of bisulfite-treated reads to a reference genome (-X 700 --dovetail). The reference genome was first transformed into bisulfite-converted version (C-to-T and G-to-A converted) and subsequently indexed using bowtie2 [47]. Sequence reads were also transformed into fully bisulfite-converted versions (C-to-T and G-to-A converted) before they were aligned to similarly converted versions of the genome in a directional manner. Sequence reads that produce a unique best alignment from the two alignment processes (original top and bottom strand) were then compared to the normal genomic sequence, and the methylation state of all cytosine positions in the read was inferred. The same reads that aligned to the same regions of genome were regarded as duplicated ones. The sequencing depth and coverage were summarized using deduplicated reads.

The results of the methylation extractor were transformed into the bigWig format for visualization using the IGV browser. The sodium bisulfite non-conversion rate was calculated as the percentage of cytosine sequenced at cytosine reference positions in the lambda genome.

To identify the methylation site, the sum Mc of methylated counts were modeled as a binomial (Bin) random variable with the methylation rate r

$$mC \sim Bln(mC+umC*r)$$

To calculate the methylation level of the sequence, the sequence was divided into multiple bins, with a bin size of 10 kb. The sum of methylated and unmethylated read counts in each window were calculated. The methylation level (ML) for each window or C site shows the fraction of methylated Cs; it was defined as:

$$ML(C) = \frac{reads\ (\mathrm{mC})}{reads\ (\mathrm{mC}) + reads\ (\mathrm{C})}$$

The calculated ML was further corrected with the bisulfite non-conversion rate according to previous studies [48]. Given the bisulfite non-conversion rate r, the corrected ML was estimated as:

$$ML_{(corrected)} = \frac{ML-r}{1-r}$$

Differentially methylated regions (DMRs) were identified using the DSS software [49] [50] [51]. According to the distribution of DMRs through the genome, the genes related to DMRs were defined as genes whose gene body region (from TSS to TES) or promoter region (upstream 2 kb from the TSS) overlap with the DMRs.

RNA sequencing (RNAseq)

Total RNA was extracted from each fresh frozen liver tissue using RNeasy Mini Kit (QIAGEN); subsequently, each library was constructed using the TruSeq RNA Sample Prep Kit v2 according to the manufacturer's instructions. RNAseq generated 100 bp paired-end sequences, and these raw reads were aligned to the reference genome sequence (http://genome.ucsc.edu/, mm10) using the Genomon RNA pipeline. Total mapped raw reads and fragments per kilobase of exons per million mapped fragments (FPKM) was calculated using the Genomon RNA pipeline [52]. Gene Set Enrichment Analysis (GSEA) was performed using public software obtained from the Broad Institute to compare the gene expression profile of each case [53].

Analysis of correlation between methylation change and expression change

The genes bearing a CG-context DMR hypomethylated in *Dnmt3b*-deficient liver were listed depending on which gene functional element they reside on. Pearson correlation coefficient was calculated between the expression fold change obtained from FPKM values in RNAseq data (N=4 for each genotype) and the length of each DMR. The total length of DMRs was used if multiple DMRs locate on one gene functional element.

Primary hepatocyte isolation

After exposure of the abdominal cavity by midline incision and placement of a 24G catheter in the inferior vena cava, the portal vein was cut and Hank's Balanced Salt Solution (HBSS) (Gibco 14170-112, Thermo Fisher Scientific, Waltham, MA) warmed up to 37°C was perfused at a rate of 10 mL/min for 5 min. Next, the liver-digesting medium, 100 mg of Collagenase type II (Gibco 17101-015) dissolved in 50 mL of HBSS, warmed at 37°C was perfused at a rate of 7 mL/min for

7 min. The whole liver was excised and minced in 20 mL of ice-cold Dulbecco's Modified Eagle Medium (DMEM) (Gibco 12800-017) + 10% Fetal Bovine Serum (FBS) (Gibco 26140-079). After filtering with a 100 µm cell strainer, the cell suspension was centrifuged at 75 × *g* for 3 min at 4°C, the supernatant was removed, and the pellet was gently resuspended in 20 mL of ice-cold DMEM + 10% FBS. This washing step was further repeated twice; finally, the pellet was resuspended in 3 mL of DMEM + 10% FBS + Penicillin-Streptomycin (Pen/Strep) (Gibco 15070-063). The cells were counted using a hematocytometer and seeded as described later in each experiment.

References for Supplementary Materials and Methods

47 Langmead, B. & Salzberg, S. L. Fast gapped-read alignment with Bowtie 2. *Nat Methods* **9**, 357-359, doi:10.1038/nmeth.1923 (2012).

48 Lister, R. *et al.* Global epigenomic reconfiguration during mammalian brain development. *Science* **341**, 1237905, doi:10.1126/science.1237905 (2013).

49 Feng, H., Conneely, K. N. & Wu, H. A Bayesian hierarchical model to detect differentially methylated loci from single nucleotide resolution sequencing data. *Nucleic Acids Res* **42**, e69, doi:10.1093/nar/gku154 (2014).

50 Wu, H. *et al.* Detection of differentially methylated regions from whole-genome bisulfite sequencing data without replicates. *Nucleic Acids Res* **43**, e141, doi:10.1093/nar/gkv715 (2015).

51 Park, Y. & Wu, H. Differential methylation analysis for BS-seq data under general experimental design. *Bioinformatics* **32**, 1446-1453, doi:10.1093/bioinformatics/btw026 (2016).

52 Sun, J., Nishiyama, T., Shimizu, K. & Kadota, K. TCC: an R package for comparing tag count data with robust normalization strategies. *BMC Bioinformatics* **14**, 219, doi:10.1186/1471-2105-14-219 (2013).

53 Subramanian, A. *et al.* Gene set enrichment analysis: a knowledge-based approach for interpreting genome-wide expression profiles. *Proc Natl Acad Sci U S A* **102**, 15545-15550, doi:10.1073/pnas.0506580102 (2005).
